# New quinazolin-2,4-dione derivatives incorporating acylthiourea, pyrazole and/or oxazole moieties as antibacterial agents *via* DNA gyrase inhibition†

**DOI:** 10.1039/d4ra02960g

**Published:** 2024-05-28

**Authors:** Amal O. A. Ibrahim, Abdelfattah Hassan, Ahmed M. Mosallam, Ahmed Khodairy, Huda R. M. Rashdan, Aboubakr H. Abdelmonsef

**Affiliations:** a Department of Chemistry, Faculty of Science, South Valley University Qena 83523 Egypt aboubakr.ahmed@sci.svu.edu.eg; b Department of Medicinal Chemistry, Faculty of Pharmacy, South Valley University Qena 83523 Egypt; c Department of Chemistry, Faculty of Science, Sohag University Sohag 82524 Egypt; d Chemistry of Natural and Microbial Products Department, Pharmaceutical and Drug Industries Research Institute, National Research Centre 33 El Buhouth St, Dokki Giza 12622 Egypt

## Abstract

This article contributes to the search for new therapeutic agents for treatment of diseases caused by bacterial pathogens. In this study, a new series of compounds incorporating numerous bioactive moieties such as quinazolin-2,4-dione, acylthiourea linkage, and/or five membered nitrogen heterocycles (pyrazole and oxazole) 2–5a–c was described to identify new antibacterial drug candidates *via* inhibition of DNA gyrase enzyme. The precursor *N*-[*N*′-(2-cyano-acetyl)-hydrazinocarbothioyl]-4-(2,4-dioxo-1,4-dihydro-2*H*-quinazolin-3-yl)-benzamide 2 was prepared by treatment of compound 1 with ammonium thiocyanate and cyanoacetic acid hydrazide through multicomponent reaction (MCR). In addition, compounds 3a–d and 4a–b were synthesized by treatment of 2 with aromatic aldehydes and/or ketones through Knoevenagel reaction, affording high purity products in satisfactory yields. Moreover, new heterocyclic moieties such as pyrazole and/or oxazole attached to quinazolin-2,4-dione core 5a–c were synthesized by treatment of 3c with different nucleophilic reagents like hydrazine, phenyl hydrazine and hydroxyl amine, respectively. Subsequently, the obtained products were structurally characterized by IR, ^1^H-, ^13^C-NMR, and MS analyses. The minimum inhibitory concentration (MIC) and antibacterial potency of all compounds were estimated against two G−ve (*E. coli* and *P. aeruginosa*), and two G+ve bacteria (*B. subtilis* and *S. aureus*). Encouragingly, compound 3c demonstrated the best antibacterial activity against all the strains of the tested pathogenic bacteria at low concentrations compared with the standard drug, Ciprofloxacin. Electron withdrawing groups such as –NO_2_ and –Cl enhance the antibacterial activity. Next, a molecular docking study between the synthesized derivatives and the target enzyme, DNA gyrase enzyme (PDB: 2xct) was undertaken to investigate intermolecular interactions between the compounds and target enzyme.

## Abstract 2

Rationale design of new quinazolindione-clubbed pyrazole and/or oxazole hybrids.

## Graphical abstract

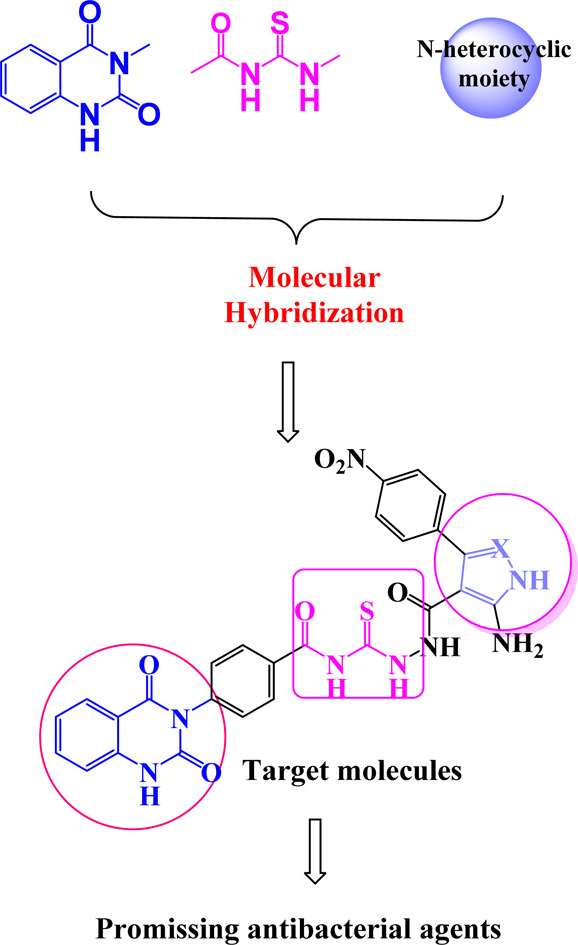

## 1. Introduction

Infectious diseases caused by bacteria pathogens, are the main cause of public health problems throughout the world.^1^ Hence, many types of drugs have been developed and used to treat various types of infections caused by bacteria.^2^ However, several reports have reported on pathogenic microorganisms that improve resistance to most available drugs.^3^ The problem is exacerbated by the rapid development of new pathogenic microorganisms.^4^ Consequently, the treatment of cancer and infectious diseases continues to be challenging at this time and requires continuous research to develop new, effective, and safe antibiotics.

**Fig. 1:**
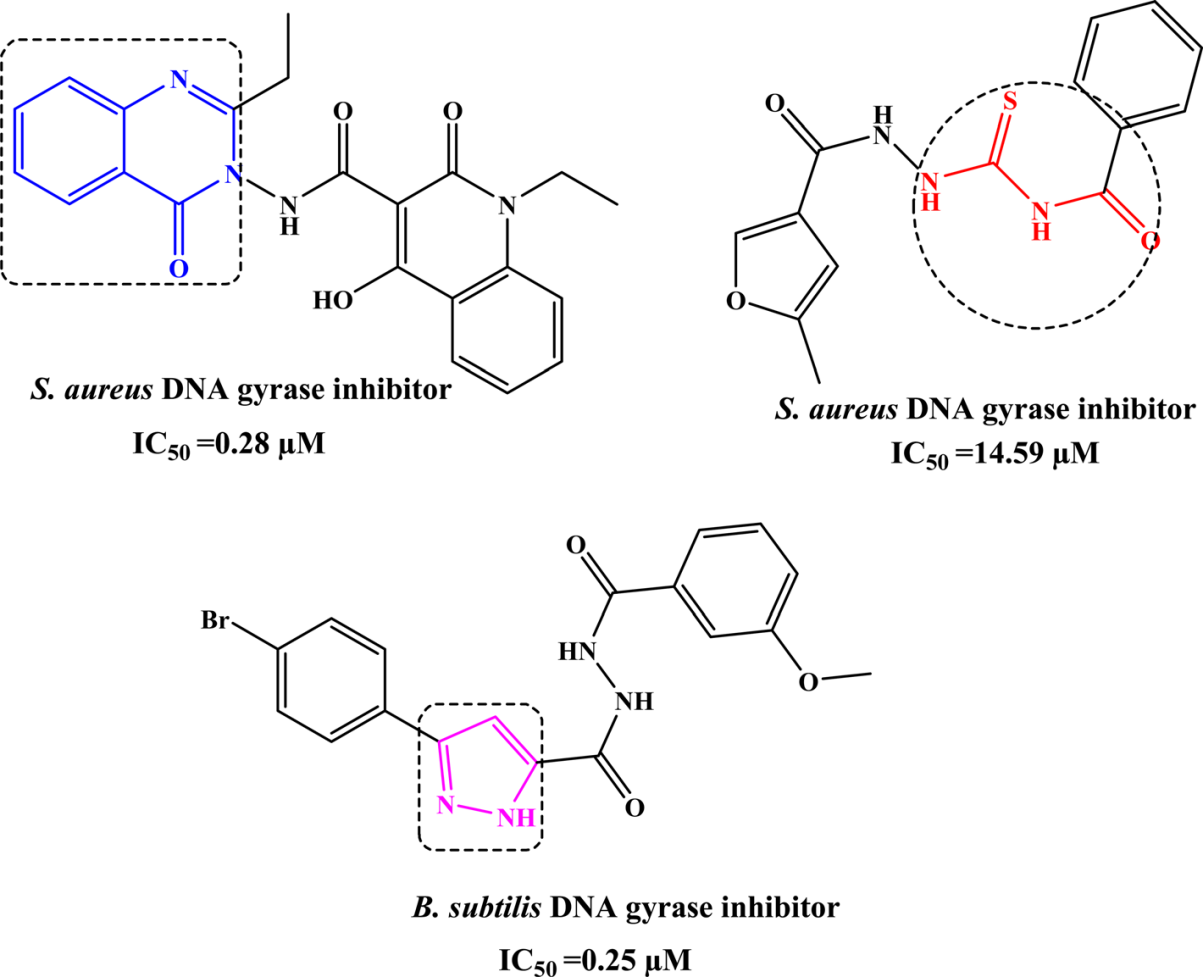
Reported quinazolinone I, acylthiourea II, and pyrazole III derivatives as antimicrobial agents targeting DNA gyrase.

By searching for antibacterial inhibitors, it was found that quinazolinone derivative I significantly inhibits the activity of *S. aureus* DNA gyrase with IC_50_ value of 0.25 µM.^5^ In addition, acylthiourea derivative II inhibits *S. aureus* DNA gyrase with IC_50_ value of 14.59 µM.^6^ Whereas, pyrazole derivative has attracted great interest due to its significant activity against *B. subtilis* DNA gyrase with IC_50_ value of 0.25 µM,^7^ as shown in Fig. 1.

**Fig. 2:**
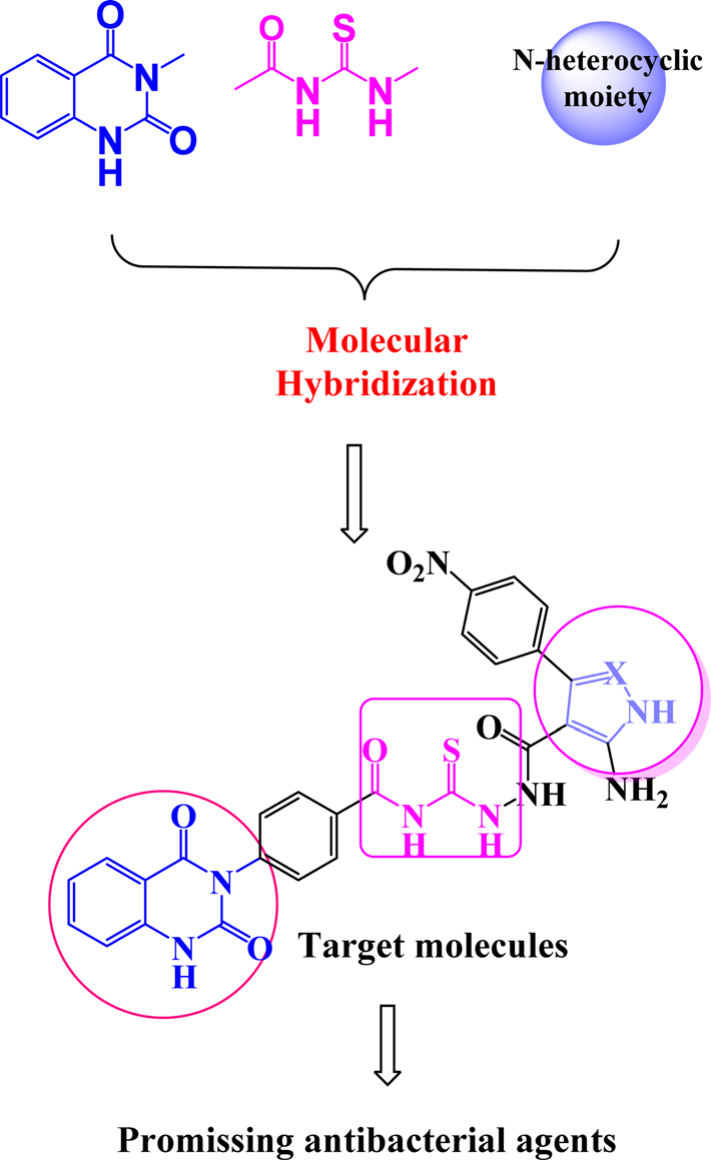
Rationale design of new quinazolindione-clubbed pyrazole and/or oxazole hybrids.

On the other hand, molecular hybridization is a rational design strategy for new ligands, based on the recognition of drug-like subunits in the molecular structure of two or more known bioactive derivatives.^8^ Even today, compounds containing quinazolin-2,4-dione scaffold^9–11^ represent an endless inspiration for the design and development of new agents showing a wide range of biological properties (Fig. 2). Acylthiourea is a functional group presents in many biologically active agents with antimicrobial, anticancer, and antioxidant^12–14^ (Fig. 2). Moreover, five membered nitrogen heterocycles are vital targets that exploited in the design of antibacterial drug candidates.^12–16^

According to the recent studies, quinazolin-2,4-dione fragment attached to acylthiourea core and/or five membered nitrogen heterocycles possess biological activity,^17^ including anticancer,^18^ anti-malarial,^19^ antibacterial,^20^ antiviral,^21^ antifungal^22^ and anti-inflammatory.^23^ Our targeted molecules structure design has created from marketed available drugs.

Inspired by the above mentioned, we aim to design new series of compounds incorporating various bioactive cores such as quinazolin-2,4-dione, acylthiourea linkage and/or five membered nitrogen heterocycles 2–5a–c as more effective DNA gyrase enzyme inhibitors, and investigate their antibacterial activities against *E. coli* and *P. aeruginosa* of two G−ve bacteria strains, and *B. subtilis* and *S. aureus* of two G+ve bacterial strains. In this study, rational approaches such as *in silico* docking study and ADMET (adsorption, distribution, metabolic, excretion, and toxicity) properties were utilized to select the best compounds to serve as potential antibacterial inhibitors.

## 2. Results and discussion

### 2.1. Chemistry

**Scheme 1:**
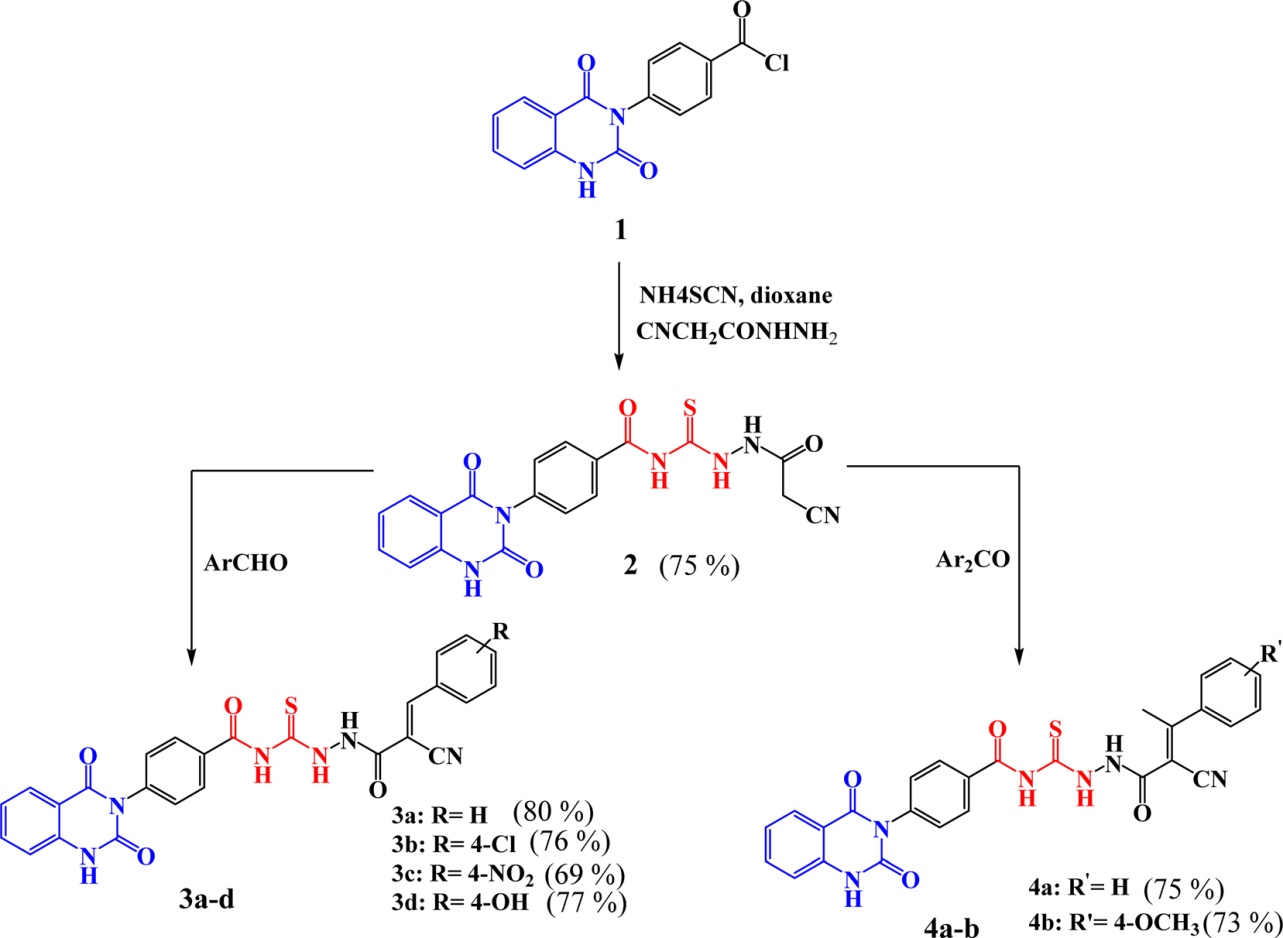
Synthetic routes of the targeted compounds 2–4a,b.

**Fig. 3:**
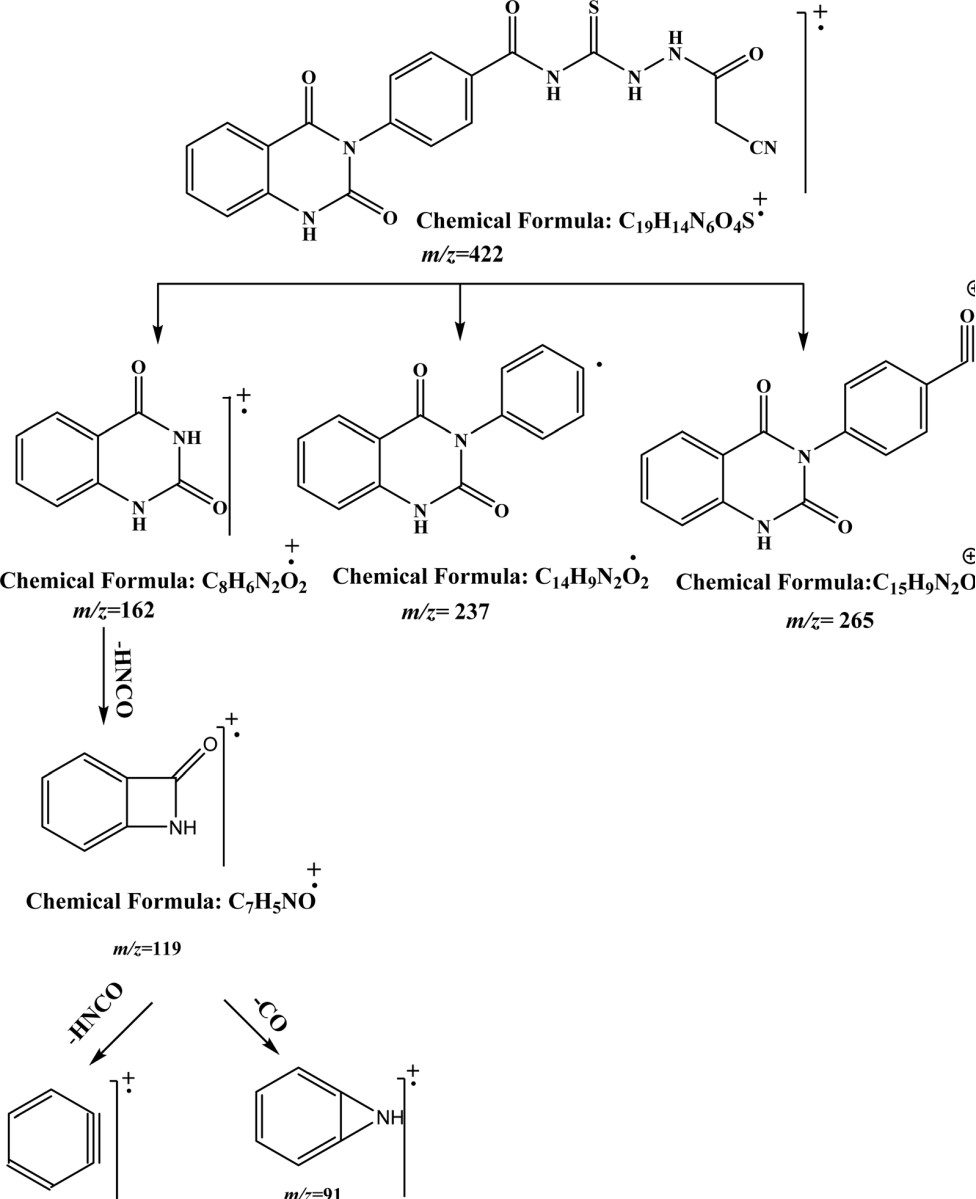
GC-MS molecular fragmentation of compound 2 and its structural visualization with mass value.

Scheme 1 outlined the synthetic pathway of derivatives 3a–d and 4a–b. Initially, precursor 2 was prepared by treatment of compound 1 with ammonium thiocyanate and cyanoacetic acid hydrazide under reflux. Its chemical structure was elucidated by the IR spectrum which showed the characteristic absorption bands at *ν* 3184, 308, 2258, 1719, and 1667 cm^−1^ characteristic to the presence of amino, cyano, and carbonyl groups, respectively. Moreover, ^1^H-NMR spectrum exhibited the presence of protons of NH groups as singlet signals at *δ* 11.63, 10.83, 10.67, 10.45 ppm, in addition to protons of methylene group appeared as singlet signal at *δ* 3.86 ppm. Further, ^13^C-NMR spectrum showed 23.6 (–CH_2_–), 114.75, 115.75, 123.06, 128.05, 128.4, 128.48, 129.72, 129.27, 129.86, 134.63, 135.77, 138.79, 150.51 (Ar–C), 162.61 (C=O), 167.97 (C=O), and 179.94 (C=S). While, the mass spectrum of compound 2 adds additional confirmation for elucidation of the chemical structure that showed molecular ion peak *m*/*z* at (422 [M]^+^) for C_19_H_14_N_6_O_4_S. Fig. 3 exhibits the structural fragmentation of the compound 2 and its mass values.

The synthesis of quinazolin-benzamide derivatives 3a–d was achieved by the reaction of compound 2 with various aromatic aldehydes such as, benzaldehyde, *p*-chlorobenzaldehyde, *p*-nitrobenzaldehyde and *p*-hydroxy benzaldehyde, respectively, through Knoevenagel reaction. The success of the formation of the new compounds 3a–d was structurally supported by spectral data. For instance, structure of compound 3a was confirmed by IR spectrum which exhibited absorption bands for NH, CN, CO and CS at *ν* 3219, 2222, 1720 and 1240 cm^−1^, respectively. However, ^1^H-NMR spectrum of 3a clearly showed the presence of NH protons as singlet signals at *δ* 11.66, 10.77, 10.75 ppm, along with aromatic protons as multiplet signals in the region of *δ* 7.24–8.33 ppm. Moreover, ^13^C-NMR spectrum decaled peaks at *δ* 112.47, 114.86, 116.95 (CN), 123.56, 128.55, 128.97, 129.62, 129.71, 129.78, 132.55, 134.63, 135.77, 138.78, 140.30, 140.35, 150.42 (Ar–C), 162.57 (C=O), 162.60 (C=O), 167.77 (C=O), 167.95 (C=O), 182.54 (C=S). Finally, the recorded mass *m*/*z* at (510 [M]^+^) which is agreement with the expected formula C_26_H_18_N_6_O_4_S.

Similarly, compounds 4a–b were synthesized in satisfactory yields with high purity by treatment of 2 with some aromatic ketones namely, acetophenone and *p*-methoxy acetophenone, through Knoevenagel reaction. Further, the characterization of compounds was investigated by their spectral data. For instance, the IR spectrum of 4b revealed absorption bands for NH, CN, CO and CS groups at *ν* 3227, 3196, 2215, 1720 and 1270 cm^−1^, respectively. The ^1^H-NMR spectrum of 4b showed singlet signals for NH protons at *δ* 11.64, 10.78, 10.67 and *δ* 10.45 ppm. Also, appearance of multiplet signals at *δ* 7.24–7.99 ppm for aromatic protons, as well as appearance of new protons of methoxy group (–OCH_3_) as a singlet signal at *δ* 3.87 ppm, beside methyl protons (–CH_3_) as a singlet signal at *δ* 3.86 ppm. Moreover, ^13^C-NMR spectrum exhibited peaks at *δ* 19.60 (–CH_3_), 46.00 (–OCH_3_), 96.40, 112.00, 114.90, 116.40 (CN), 117.30, 127.50, 127.80, 127.90, 128.20, 128.30, 128.40, 129.00, 133.60, 134.80, 135.40, 139.70, 150.0, 161.20 (C=O), 162.10 (C=O), 167.70 (C=O), 168.70 (C=O), 179.90 (C=S). Whereas the mass spectrum of the obtained compound adds additional confirmation for elucidation of the chemical structure that showed molecular ion peak *m*/*z* at (554 [M]^+^) for C_28_H_22_N_6_O_5_S.

**Scheme 2:**
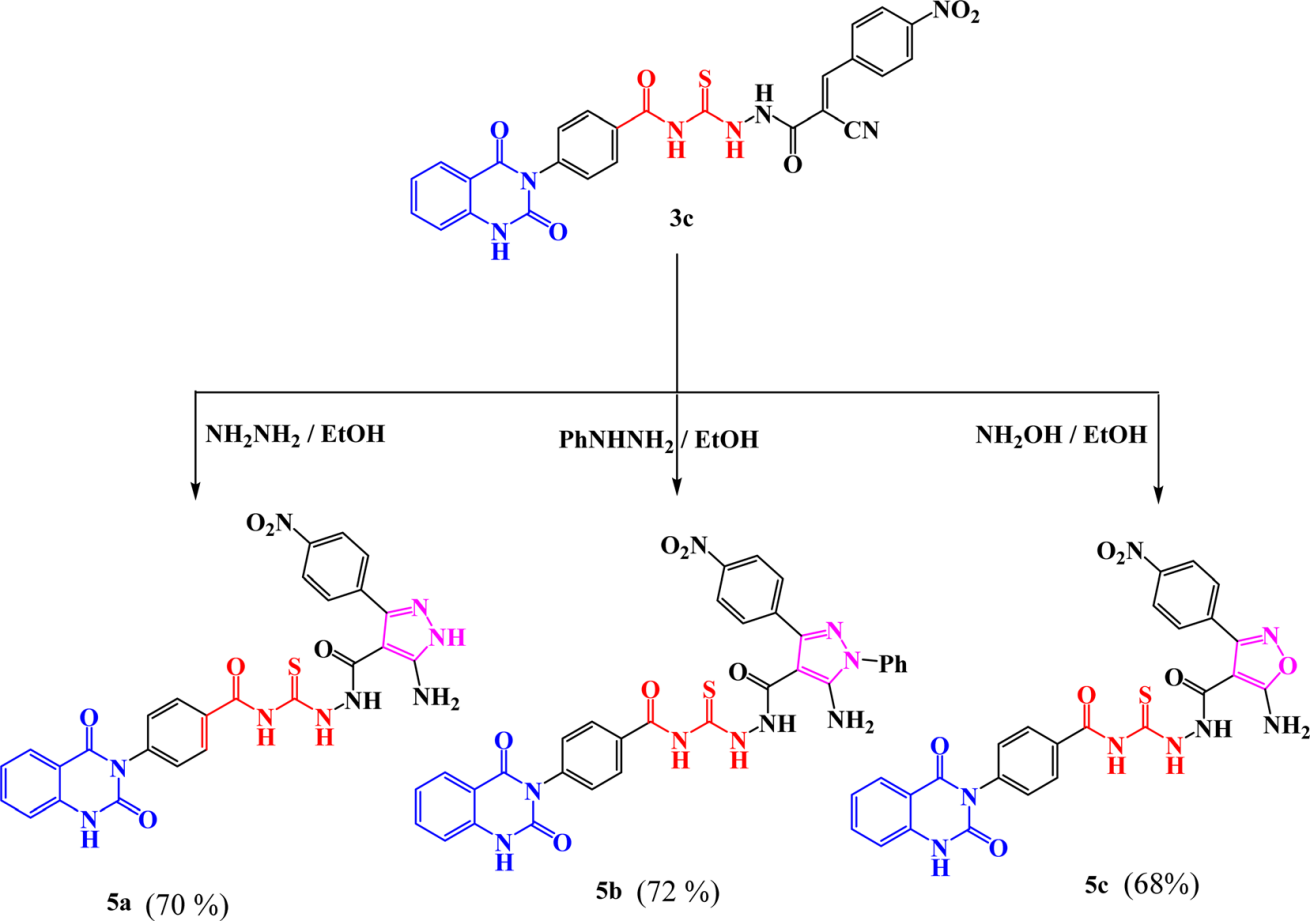
Synthesis of compounds 5a–c.

**Scheme 3:**
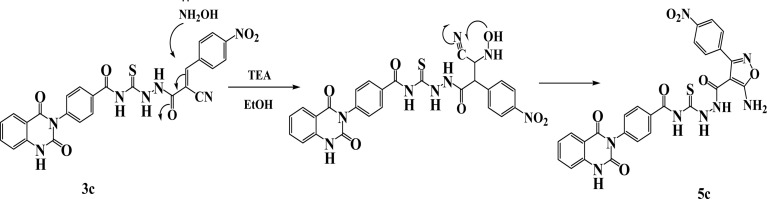
Synthetic pathway of compounds 5c.

New compounds incorporating quinazolin-2,4-dione attached to N-heterocyclic moieties such as pyrazole and/or oxazole through acylthiourea linkage 5a–c were prepared by the reaction of 3c with various nitrogen nucleophiles such as hydrazine, phenyl hydrazine and hydroxyl amine through nucleophilic addition reaction, as shown in Scheme 2. Further, the structure elucidation of compounds was investigated by their spectral data. For instance, the IR spectrum of compound 5c illustrated the presence of NH_2_, NH, C=O's and C=S at 3209, 3115, 1717, 1670 and 1271 cm^−1^, respectively. ^1^H-NMR spectrum of compound 5c exhibited the presence of proton of NH groups as singlet signals at *δ* 11.64, 11.60, 10.63, 9.92 ppm, in addition to protons of amino group appeared as singlet signal at *δ* 6.29 ppm. While ^13^C-NMR spectrum showed *δ* 114.76, 115.75, 123.06, 128.05, 128.47, 129.57, 129.69, 129.80, 129.99, 130.11, 130.28, 131.04, 134.64, 135.77, 138.78, 140.30, 150.50 (Ar–C), 162.56 (C=O), 162.60 (C=O), 167.35 (C=O), 167.94 (C=S). The suggested mechanism for preparation of compound 5c is represented in Scheme 3. The spectral analyses of the synthesized compounds are included in the ESI as Fig. S1–S39.†

### 2.2. Antibacterial activity

The global significance of antibiotic resistance as a severe danger to public health, resulting in diminished effectiveness of antibiotics, has been well acknowledged. Therefore, the development of novel medication candidates with broad-spectrum antibacterial properties could help address these difficulties. In this study, the antibacterial efficacy of the prepared compounds 2–4b toward various pathogenic microbes was estimated. The MIC was stated, as represented in Table 1.

**Table 1:** The MIC of the newly prepared compounds^a^

Sample no.	(MIC, µM)			
	*E. coli*	*P. aeruginosa*	*B. subtilis*	*S. aureus*
2	0.049	0.047	0.284	0.094
3a	ND	ND	ND	ND
3b	0.073	0.110	ND	ND
3c	0.004	0.009	0.018	0.009
3d	ND	ND	ND	ND
4a	0.019	0.009	0.019	0.013
4b	0.018	ND	0.288	0.144
5a	ND	ND	ND	ND
5b	ND	ND	ND	ND
5c	ND	ND	ND	ND
Ciprofloxacin	5	7	2.5	1.25

a The standard drug is Ciprofloxacin, ND: not determined.

It was noticed that the tested compounds revealed a considerable wide broad spectrum of antibacterial potency against most of the strains of the tested pathogenic microbes. Meanwhile, 3c exhibited the most remarkable antibacterial efficacy toward all the strains of the tested pathogenic bacteria at lower concentrations than the reference drug, ciprofloxacin.

### 2.3. *In silico* molecular docking studies

**Fig. 4:**
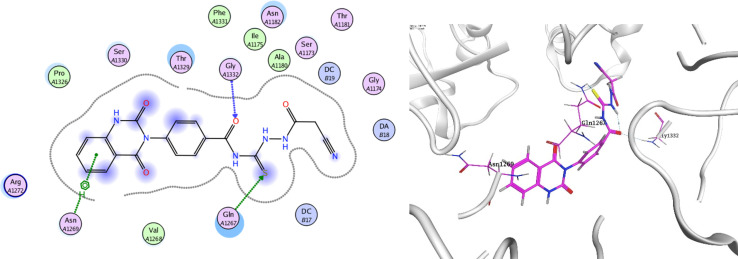
Docking style of compound 2 with DNA gyrase (PDB: 2xct).

**Fig. 5:**
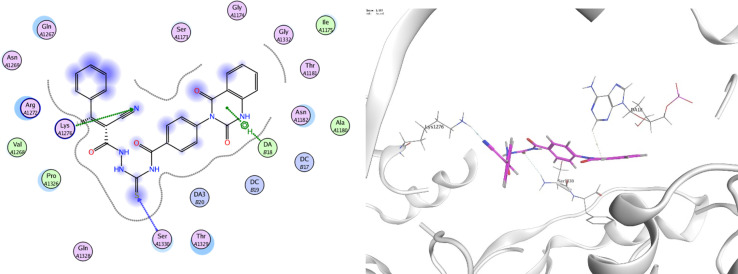
Docking style of compound 3a with DNA gyrase (PDB: 2xct).

**Fig. 6:**
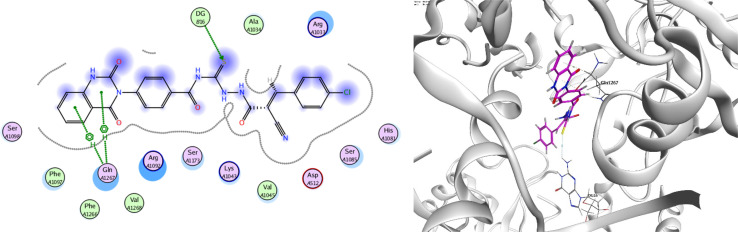
Docking style of compound 3b with DNA gyrase (PDB: 2xct).

**Fig. 7:**
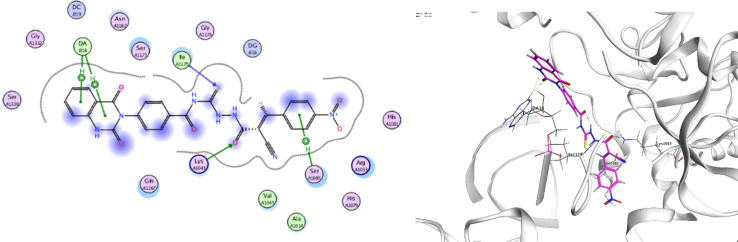
Docking style of compound 3c with DNA gyrase (PDB: 2xct).

**Fig. 8:**
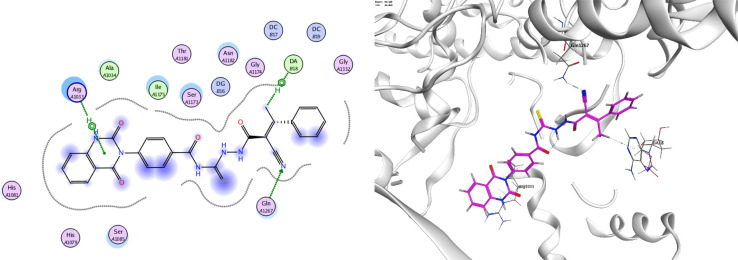
Docking style of compound 4a with DNA gyrase (PDB: 2xct).

**Fig. 9:**
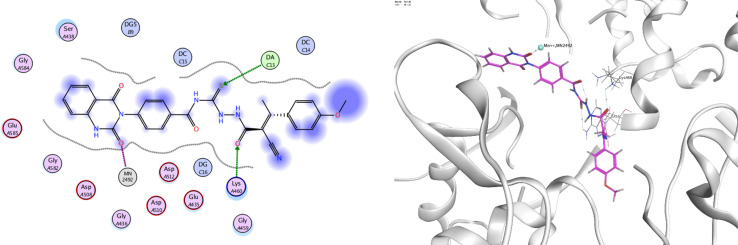
Docking style of compound 4b with DNA gyrase (PDB: 2xct).

In order to investigate the molecular mechanism of the antibacterial action of the target compounds, we performed molecular docking experiments,^24^ using the MOE program. The molecular docking tests demonstrated favorable interactions between the synthesized derivatives and the target protein, DNA gyrase enzyme (PDB: 2xct).^25^Fig. 4–9 exhibited the 2D and 3D representations of docking styles of target compounds 2, 3a–c and 4a,b with active site of DNA gyrase enzyme. Compound 2 formed two HB with Gly1332 and Gln1267 *via* carbonyl oxygen and thiourea sulfur atoms, respectively. Additionally, quinazoline ring of compound 2 made pi–H with Asn1269 (Fig. 4). Compound 3a formed dual HB with Lys1276 and Ser1330 *via* nitrile nitrogen and thiourea sulfur atoms, respectively. Moreover, the hetero ring of quinazoline of compound 3a interacts with adenine DA18 (Fig. 5). Compound 3b formed HB with guanine DG16 through thiourea group and dual pi–H interactions with Gln1267 through quinazoline moiety (Fig. 6). Out of all target compounds, 3c showed intricate interactions with DNA gyrase which aligns with its high antimicrobial activity. Compound 3c formed two HB with Lys1043 and Ile1175 *via* carbonyl and thiourea groups, respectively (Fig. 7). In addition, the quinazoline ring of compound 3c had dual pi–H interactions with adenine DA18 and another pi–H interaction between distal phenyl ring and Ser1085. Compound 4a formed HB with Gln1267 and two pi–H interactions with Arg1033 and adenine DA18 (Fig. 8). Finally, 2-oxo moiety of quinazolin-2,4-dione of compound 4b interact with magnesium MN2492 (Fig. 9). Also, compound 4b formed two HB with Lys460 and adenine DA13.

**Fig. 10:**
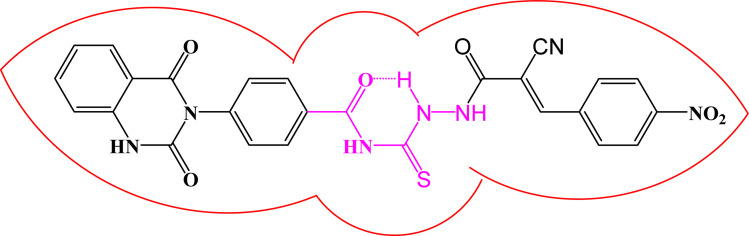
Intramolecular HB of compound 3c.

One of characteristic structural features of target compounds is their ability to form stable intramolecular HB between carbonyl oxygen and hydrogen of thiourea moiety which results in a highly stable classical pseudo six-membered ring. This intramolecular HB was clear in the most stable poses of compounds 2, 3c, 4a and 4b. Along with insertion of unsaturation, intramolecular HB is considered one of the skillful rigidification strategies in drug design. Thanks to the intramolecular HB bond, compound 3c, for example, adopts more extended conformation that enhances its interactions with bonding site of DNA gyrase (Fig. 10).

### 2.4 Physicochemical and pharmacokinetics prediction

In order to reach the clinic, potential drug candidate must exhibit a reasonable pharmacokinetic profile. Consequently, the physicochemical and pharmacokinetic characteristics of the target derivatives were forecasted *via* SwissADME, as illustrated in Tables 2–4. All target compounds were predicted not to cause centrally adverse effects as they predicted not to pass blood brain barrier. All target compounds were predicted to be resistant to P-gp efflux. The expected impact of the target drugs on CYP450 enzymes, namely CYP1A2, CYP2C19, CYP2C9, CYP2D6, and CYP3A4, suggests that there is a low likelihood of drug–drug interactions occurring. All target compounds were expected not to be inhibitors for all mentioned CYP enzymes except CYP2C9 and CYP3A4.

**Table 2:** Physicochemical properties of target compounds and ciprofloxacin^a^

Molecule	MW	#Heavy atoms	#Aromatic heavy atoms	Fraction Csp^3^	#Rotatable bonds	#HBAs	#HBDs	MR	TPSA
2	422.42	30	16	0.05	8	5	4	110.93	180.97
3a	510.52	37	22	0	9	5	4	140.54	180.97
3b	544.97	38	22	0	9	5	4	145.55	180.97
3c	555.52	40	22	0	10	7	4	149.36	226.79
3d	526.52	38	22	0	9	6	5	142.56	201.2
4a	524.55	38	22	0.04	9	5	4	145.35	180.97
4b	554.58	40	22	0.07	10	6	4	151.84	190.2
5a	585.55	42	27	0	10	7	6	157.08	257.7
5b	661.65	48	33	0	11	7	5	182.06	246.84
5c	586.54	42	27	0	10	8	5	155	255.05
Ciprofloxacin	331.34	24	10	0.41	3	5	2	95.25	74.57

a HBAs: hydrogen bond acceptors, HBDs: hydrogen bond donors, MR: molar refractivity, TPSA: topological polar surface area.

**Table 3:** Pharmacokinetics of target compounds and ciprofloxacin

Molecule	BBB permeant	Pgp substrate	CYP1A2 inhibitor	CYP2C19 inhibitor	CYP2C9 inhibitor	CYP2D6 inhibitor	CYP3A4 inhibitor
2	No	No	No	No	No	No	No
3a	No	No	No	No	Yes	No	Yes
3b	No	No	No	No	Yes	No	Yes
3c	No	No	No	No	No	No	Yes
3d	No	No	No	No	No	No	Yes
4a	No	No	No	No	Yes	No	Yes
4b	No	No	No	No	Yes	No	Yes
5a	No	No	No	No	No	No	Yes
5b	No	No	No	No	No	No	Yes
5c	No	No	No	No	No	No	Yes
Ciprofloxacin	No	Yes	No	No	No	No	No

**Table 4:** Drug likeness parameters of target compounds and ciprofloxacin

Molecule	Lipinski #violations	Ghose #violations	Veber #violations	Egan #violations	Muegge #violations	Bioavailability score
2	0	0	1	1	1	0.55
3a	1	2	1	1	1	0.55
3b	1	2	1	1	1	0.55
3c	2	2	1	1	1	0.17
3d	2	2	1	1	1	0.17
4a	1	2	1	1	1	0.55
4b	2	2	1	1	1	0.17
5a	3	2	1	1	2	0.17
5b	2	3	2	1	3	0.17
5c	2	2	1	1	1	0.17
Ciprofloxacin	0	0	0	0	0	0.55

Compound 2 has a molecular weight of less than 500 without any Lipinski violations. The rest of target compounds have molecular weights 510–661 g mol^−1^ which is considered the only Lipinski violation of compounds 3a, 3b and 4a. Compounds 3c, 3d, 4b, 5b and 5c have addition Lipinski violation because nitrogen and oxygen more than ten atoms. All target compounds have only one violation in Egan filter. All compounds have no violations in Muegge filter except compounds 5a and 5b. In relation to the Abbott bioavailability score, it was observed that target compounds 2, 3a, and 3b exhibit oral absorption comparable to that of ciprofloxacin.

## 3. Experimental

### 3.1. Chemistry

Solvents and reagents were obtained from commercial sources. Melting points were uncorrected and detected on electrothermal apparatus and. Infrared (IR) spectra were recorded on a Shimadzu 8101 PC spectrometer. ^1^H- and ^13^C-NMR spectra were carried out on a Varian Mercury 400, and 100 MHz spectrophotometer using DMSO-d_6_ as a solvent. Electron impact mass spectra were obtained at 70 eV using a GCMS-QP 1000 EX spectrometer.

#### 3.1.1. Synthesis of 4-(2,4-dioxo-1,4-dihydro-2*H*-quinazolin-3-yl)-benzoyl chloride 1

Compound 1 was synthesized by the reported method.^11,21^

#### 3.1.2. Synthesis of *N*-[*N*′-(2-cyano-acetyl)-hydrazino-carbo-thioyl]-4-(2,4-dioxo-1,4-dihydro-2*H*-quinazolin-3-yl)-benzamide 2

Cyanoacetic acid hydrazide (0.003 mol, 0.33 g) in dioxane (20 ml) was added to a mixture of 1 (0.003 mol, 1 g) and NH_4_SCN (0.003 mol, 0.32 g) in dioxane (15 ml), then the whole mixture was refluxed for 2 h. After completion of the reaction, the solid formed was collected and recrystallized from dioxane/ethanol to yield the desired compound 2 as white powder. Yield: 75%. M.P.: >300 °C. IR = 3184, 3085 (NH), 2258 (CN), 1719, 1667 (CO), 1282 (CS). ^1^H-NMR: *δ* 10.45–11.63 (s, 4H, 4NH), 7.25–7.97 (m, 8H, Ar–H), 3.86 (s, 2H, CH_2_). ^13^C-NMR: *δ* 23.6 (–CH_2_–), 114.75, 115.75, 123.06, 128.05, 128.4, 128.48, 129.72, 129.27, 129.86, 134.63, 135.77, 138.79, 150.51 (Ar–C), 162.61 (C=O), 167.97 (C=O), and 179.94 (C=S). MS (El): *m*/*z* (%) = 422 [M]^+^ and 425 [M]^+^ + 3. Anal. calcd for C_19_H_14_N_6_O_4_S: C, 54.02; H, 3.34; N, 19.90; S, 7.59%, found C, 53.95; H, 3.48; N, 19.83; S, 7.68%.

#### 3.1.3. Synthesis of arylquinazolin-2,4-diones 3a–d

Treatment of precursor 2 with aromatic aldehydes namely, benzaldehyde, *p*-chlorobenzaldehyde, *p*-nitrobenzaldehyde and/or *p*-hydroxy benzaldehyde in ethanol (25 ml) and few drops of piperidine under reflux for 8–10 h yielded 3a–d, respectively.

#### 3.1.4. Synthesis of *N*-(2-(2-cyano-3-phenylacryloyl)-hydrazine-1-carbono-thioyl)-4-(2,4-dioxo-1,4-dihydroquinazolin-3(2*H*)-yl)-benzamide 3a

Dark brown powder. Yield 80%. MP: 210–212 °C. IR = 3219 (NH), 2222 (CN), 1720, 1664 (CO), 1240 (CS). ^1^H-NMR: *δ* 10–.75–11.66 (s, 3H, 3NH), 7.24–8.33 (m, 14H, Ar–H + CH). ^13^C-NMR: *δ* 112.47, 114.86, 116.95 (CN), 123.56, 128.55, 128.97, 129.62, 129.71, 129.78, 132.55, 134.63, 135.77, 138.78, 140.30, 140.35, 150.42 (Ar–C), 162.57 (C=O), 162.60 (C=O), 167.77 (C=O), 167.95 (C=O), 182.54 (C=S). MS (El): *m*/*z* (%) = 510 [M]^+^. Anal. calcd for C_26_H_18_N_6_O_4_S: C, 61.17; H, 3.55; N, 16.46; S, 6.28%, found: C, 61.56; H, 3.89; N, 16.06; S, 6.49%.

#### 3.1.5. Synthesis of *N*-(2-(3-(4-chlorophenyl)-2-cyanoacryloyl)-hydrazine-1-carbonothioyl)-4-(2,4-dioxo-1,4-dihydro-quinazolin-3(2*H*)-yl)-benzamide 3b

Pale yellow powder. Yield: 76%. M.P.: 225–227 °C. IR = 3193 (NH), 2221 (CN), 1724, 1670 (CO), 1269 (CS). ^1^H-NMR *δ*: 11.62–11.64 (s, 2H, 2NH), 7.25–8.05 (m, 13H, Ar–H + CH). ^13^C-NMR: *δ* 111.50, 112.05, 114.86, 115.85 (CN), 123.96, 128.95, 129.47, 129.52, 129.81, 129.88, 134.83, 135.78, 138.98, 140.31, 150.50, 158.47 (Ar–C), 162.31 (C=O), 162.60 (C=O), 167.94 (C=O), 168.84 (C=O), 182.90 (C=S). MS (El): *m*/*z* (%) = 544 [M]^+^, 546 [M]^+^ + 2. Anal. calcd for C_26_H_17_ClN_6_O_4_S: C, 57.30; H, 3.14; Cl, 6.50; N, 15.42; S, 5.88%, found: C, 57.45; H, 3.27; Cl, 6.66; N, 15.31; S, 5.98%.

#### 3.1.6. Synthesis of *N*-(2-(2-cyano-3-(4-nitrophenyl)-acryloyl)-hydrazine-1-carbonothioyl)-4-(2,4-dioxo-1,4-dihydroquinazolin-3(2*H*)-yl)benzamide 3c

Orange crystals. Yield: 69%. M.P.: 292–294 °C. IR = 3215 (NH), 2209 (CN), 1729, 1650 (CO), 1239 (CS).^1^H-NMR: *δ* 11.62–11.65 (s, 3H, 3NH), 7.25–8.31 (m, 13H, Ar–H + C–H). MS (El): *m*/*z* (%) = 555 [M]^+^. Anal. calcd for C_26_H_17_N_7_O_6_S: C, 56.21; H, 3.08; N, 17.65; S, 5.77%, found: C, 56.45; H, 3.22; N, 17.49; S, 5.89%.

#### 3.1.7. Synthesis of *N*-(2-(2-cyano-3-(4-hydroxyphenyl)-acryloyl)-hydrazine-1-carbonothioyl)-4-(2,4-dioxo-1,4-dihydro-quinazolin-3(2*H*)-yl)-benzamide 3d

Yellow crystals. Yield: 77%. M.P.: 278–280 °C. IR = 3280 (OH), 3000 (NH), 2218 (CN), 1721, 1672 (CO), 1270 (CS). ^1^H-NMR: *δ* 11.62–11.64 (s, 2H, 2NH), 7.25–7.99 (m, 13H, Ar–H + CH). MS (El): *m*/*z* (%) = 526 [M]^+^. Anal. calcd for C_26_H_18_N_6_O_5_S: C, 59.31; H, 3.45; N, 15.96; S, 6.09%, found: C, 59.45; H, 3.60; N, 15.84; S, 6.11%.

#### 3.1.8. Synthesis of arylquinazolin-2,4-diones 4a–b

Reaction of precursor 2 with aromatic ketones namely acetophenone and/or *p*-methoxy acetophenone in ethanol (25 ml) and few drops of piperidine under reflux for 8–10 h afforded compounds 4a–b, respectively.

#### 3.1.9. Synthesis of *N*-(2-(2-cyano-3-phenylbut-2-enoyl) hydrazine-1-carbonothioyl)-4-(2,4-dioxo-1,4-dihydroquinazolin-3(2*H*)-yl)benzamide 4a

Dark brown crystals. Yield: 75%. M.P.: < 300 °C. IR = 3230, 3190 (NH's), 2218 (CN), 1722, 1636 (CO), 1265 (CS). ^1^H-NMR: *δ* 10.45–11.64 (s, 4H, 4NH), 7.24–7.99 (m, 12H, Ar–H), 3.86 (s, 3H, CH_3_). ^13^C-NMR: *δ* 18.72 (–CH_3_), 95.63, 111.16, 114.78, 115.78 (CN), 120.02, 123.08, 128.06, 128.87, 129.80, 130.59, 134.96, 135.81, 139.36, 140.34, 144.07, 150.49, 162.62, 165.84 (C=O), 166.16 (C=O), 167.84 (C=O), 168.16 (C=O), 182.06 (C=S). MS (El): *m*/*z* (%) = 524 [M]^+^. Anal. calcd for C_27_H_20_N_6_O_4_S: C, 61.82; H, 3.84; N, 16.02; S, 6.11%, found: C, 62.93; H, 3.97; N, 15.88; S, 6.21%.

#### 3.1.10. Synthesis of *N*-(2-(2-cyano-3-(4-methoxyphenyl)but-2-enoyl) hydrazine-1-carbonothioyl)-4-(2,4-dioxo-1,4-dihydroquinazolin-3(2*H*)-yl)benzamide 4b

Yellowish brown powder. Yield: 73%. M.P.: >300 °C. IR = 3227, 3196 (NH's), 2215 (CN), 1720, 1665 (C=O's), 1270 (C=S). ^1^H-NMR: *δ* 10.43–11.64 (s, 4H, 4NH), 7.24–7.99 (m, 12H, Ar–H), 3.87 (s, 3H, OCH_3_), 3.86 (s, 3H, CH_3_). ^13^C-NMR: *δ* 19.60 (–CH_3_), 46.00 (–OCH_3_), 96.40, 112.00, 114.90, 116.40 (CN), 117.30, 127.50, 127.80, 127.90, 128.20, 128.30, 128.40, 129.00, 133.60, 134.80, 135.40, 139.70, 150.0, 161.20 (C=O), 162.10 (C=O), 167.70 (C=O), 168.70 (C=O), 179.90 (C=S). MS (El): *m*/*z* (%) = 554 [M]^+^. Anal. calcd for C_28_H_22_N_6_O_5_S: C, 60.64; H, 4.00; N, 15.15; S, 5.78%, found: C, 60.77; H, 4.12; N, 15.03; S, 5.90%.

#### 3.1.11. General procedures for the synthesis of compounds 5a–c

Reaction of *N*-(2-(2-cyano-3-(4-nitrophenyl)-acryloyl)-hydrazine-1-carbonothioyl)-4-(2,4-dioxo-1,4-dihydroquinazolin-3(2*H*)-yl)benzamide 3c with hydrazine hydrate, phenyl hydrazine and/or hydroxyl amine hydrochloride in ethanol (25 ml) and few drops of TEA under reflux for 8–10 h afforded compounds 5a–c, respectively.

#### 3.1.12. *N*-(2-(5-Amino-3-(4-nitrophenyl)-1*H*-pyrazole-4-carbonyl)-hydrazine-1-carbonothioyl)-4-(2,4-dioxo-1,4-dihydroquinazolin-3(2*H*)-yl)benzamide 5a

Orange crystals. Yield: 70%. M.P.: 295–297 °C. IR = 3201 (NH_2_), 2925 (NH), 1717, 1667 (C=O's), 1273 (C=S). ^1^H-NMR: *δ* 11.64 (s, 1H, NH), 11.36 (s, 1H, NH), 9.91 (s, 1H, NH), 9.64 (s, 1H, NH), 7.43–8.47 (m, 12H, Ar–H), 6.66 (s, 2H, NH_2_). ^13^C-NMR: *δ* 112.82, 114.84, 115.88, 120.22, 123.28, 125.11, 128.06, 128.87, 129.80, 130.69, 134.26, 135.83, 138.78, 139.33, 140.28, 145.17, 150.49 (Ar–C), 163.12 (C=O), 166.04 (C=O), 166.16 (C=O), 167.49 (C=S). MS (El): *m*/*z* (%) = 585 [M]^+^. Anal. calcd for C_26_H_19_N_9_O_6_S: C, 53.33; H, 3.27; N, 21.53; S, 5.48%, found: C, 53.48; H, 3.32; N, 21.44; S, 5.62%.

#### 3.1.13. *N*-(2-(5-Amino-3-(4-nitrophenyl)-1-phenyl-1*H*-pyrazole-4-carbonyl)-hydrazine-1-carbon thioyl)-4-(2,4-dioxo-1,4-dihydroquinazolin-3(2*H*)-yl)benzamide 5b

Grey crystals. Yield: 72%. M.P.: 298–300 °C. IR = 3380 (NH_2_), 2924 (NH), 1717, 1670 (C=O's), 1248 (C=S). ^1^H-NMR: *δ* 11.67 (s, 1H, NH), 11.61 (s, 1H, NH), 11.55 (s, 1H, NH), 10.89 (s, 1H, NH), 6.53–8.46 (m, 17H, Ar–H), 6.09 (s, 2H, NH_2_). ^13^C-NMR: *δ* 100.56, 100.57, 114.77, 115.78, 123.09, 124.35, 128.05, 128.75, 129.71, 129.78, 129.86, 132.55, 135.82, 140.32, 140.35, 150.42, 157.76, 158.05, 162.57 (Ar–C), 165.78, 165.79 (C=O), 167.76 (C=O), 167.77 (C=O), 182.54 (C=S). MS (El): *m*/*z* (%) = 661 [M]^+^. Anal. calcd for C_32_H_23_N_9_O_6_S: C, 58.09; H, 3.50; N, 19.05; S, 4.85%, found: C, 59.01; H, 3.61; N, 18.89; S, 4.94%.

#### 3.1.14. *N*-(2-(5-Amino-3-(4-nitrophenyl)-isoxazole-4-carbonyl)-hydrazine-1-carbonothioyl)-4-(2,4-dioxo-1,4-dihydroquinazolin-3(2*H*)-yl)-benzamide 5c

Yellowish brown crystals. Yield: 68%. M.P.: >300 °C. IR = 3209 (NH_2_), 3115 (NH), 1717, 1670 (C=O's), 1271 (C=S). ^1^H-NMR: *δ* 11.64 (s, 1H, NH), 11.60 (s, 1H, NH), 10.36 (s, 1H, NH), 9.92 (s, 1H, NH), 7.06–8.47 (m, 12H, Ar–H), 6.29 (s, 2H, NH_2_). ^13^C-NMR: *δ* 114.76, 115.75, 123.06, 128.05, 128.47, 129.57, 129.69, 129.80, 129.99, 130.11, 130.28, 131.04, 134.64, 135.77, 138.78, 140.30, 150.50 (Ar–C), 162.56 (C=O), 162.60 (C=O), 167.35 (C=O), 167.94 (C=S). MS (El): *m*/*z* (%) = 586 [M]^+^. Anal. calcd for C_26_H_18_N_8_O_7_S: C, 53.24; H, 3.09; N, 19.10; S, 5.47%, found: C, 53.33; H, 3.14; N, 19.01; S, 5.55%.

### 3.2 Antibacterial potency

The minimum inhibitory concentrations (MICs) of the prepared derivatives were estimated against *E. coli* and *P. aeruginosa* of two G−ve bacteria strains, and *B. subtilis* and *S. aureus* of two G+ve bacteria strains. The pathogens under study were provided by Al-Azhar University, Egypt. They were cultivated in Mueller Hinton broth at 35 ± 2 °C for 24 h. The antimicrobial activity and MIC were carried out as described by Qader *et al.* (2021).^26^

### 3.3 *In silico* molecular docking studies

Molecular docking studies on the synthesized compounds were performed using MOE software developed by the Chemical Computing Group ULC, Montreal, Canada. Also, the representation 2D style of the interactions between the synthesized compounds and the target protein was performed using MOE software.

## 4. Conclusion

This study involved the design, and synthesis of a novel set of hybrid compounds (2, 3a–d, 4a–b and 5a–c) incorporating quinazolin-2,4-dione analogue, acylthiourea core and/or five membered nitrogen heterocycles. The objective was to assess their potential as antibacterial agents. The *in vitro* investigations predominantly demonstrated that compound 3c (with electron withdrawing group –NO_2_) displayed the highest antibacterial efficacy against all tested harmful bacteria strains at low doses, surpassing the conventional medication Ciprofloxacin. The results were also correlated with the molecular docking investigations, which determined that compound 3c exhibited significant inhibitory activity against the target protein, DNA gyrase enzyme (PDB: 2xct). Thus, it can serve as drug candidate to develop more potent antibacterial agents due to its high inhibition activity against DNA gyrase enzyme.

## Conflicts of interest

There are no conflicts to declare.

## Supplementary Material

RA-014-D4RA02960G-s001
