# Supplementary material for: New quinazolin-2,4-dione derivatives incorporating acylthiourea, pyrazole and/or oxazole moieties as antibacterial agents via DNA gyrase inhibition
Source: RSC Adv. 2024 May 28;14(24):17158–69. doi: 10.1039/d4ra02960g (PMC11130761; doi:10.1039/d4ra02960g)
Supplement: RA-014-D4RA02960G-s001 [file RA-014-D4RA02960G-s001.zip › supplementary_material_ra-art-04-2024-002960r1-15-5-20241.docx]

**New Quinazolin-2,4-dione Derivatives Incorporating Acylthiourea, Pyrazole and/or Oxazole Moieties as Antibacterial Agents via DNA gyrase Inhibition**

Amal O. A. Ibrahim^1^, Abdelfattah Hassan^2^, Ahmed M. Mosallam^1^, Ahmed Khodairy^3^, Huda R. M. Rashdan^4^, Aboubakr H. Abdelmonsef ^1,^*

^1^Department of Chemistry, Faculty of Science, South Valley University, Qena 83523, Egypt

^2^Department of Medicinal Chemistry, Faculty of Pharmacy, South Valley University, Qena 83523, Egypt

^3^Department of Chemistry, Faculty of Science, Sohag University, Sohag 82524, Egypt

^4^Chemistry of Natural and Microbial Products Department, Pharmaceutical and Drug Industries Research Institute, National Research Centre, 33 El Buhouth St, Dokki, Giza 12622, Egypt

**List of Figures**

**Figure S1: IR spectrum of compound 1............................................................................. 1**

**Figure S2: ^1^H-NMR spectrum of compound 1................................................................... 2**

**Figure S3: ^13^C-NMR spectrum of compound 1................................................................ 3**

**Figure S4: Mass spectrum of compound 1........................................................................ 4**

**Figure S5: IR spectrum of compound 2............................................................................ 5**

**Figure S6: ^1^H-NMR spectrum of compound 2.................................................................. 6**

**Figure S7: ^13^C-NMR spectrum of compound 2................................................................ 7**

**Figure S8: Mass spectrum of compound 2.......................................................................... 8**

**Figure S9: IR spectrum of compound 3a. .......................................................................... 9**

**Figure S10: ^1^H-NMR spectrum of compound 3a. ..............................................................10**

**Figure S11: ^13^C-NMR spectrum of compound 3a ..............................................................11**

**Figure S12: Mass spectrum of compound 3a....................................................................12**

**Figure S13: IR spectrum of compound 3b........................................................................ 13**

**Figure S14: ^1^H-NMR spectrum of compound 3b............................................................... 14**

**Figure S15: ^13^C-NMR spectrum of compound 3b ...............................................................15**

**Figure S16: Mass spectrum of compound 3b................................................................... 16**

**Figure S17: IR spectrum of compound 3c ....................................................................... 17**

**Figure S18: ^1^H-NMR spectrum of compound 3c. .............................................................. 18**

**Figure S19: Mass spectrum of compound 3c.................................................................... 19**

**Figure S20: IR spectrum of compound 3d.......................................................................... 20**

**Figure S21: ^1^H-NMR spectrum of compound 3d................................................................ 21**

**Figure S22: Mass spectrum of compound 3d. .................................................................. 22**

**Figure S23: IR spectrum of compound 4a. ........................................................................ 23**

**Figure S24: ^1^H-NMR spectrum of compound 4a............................................................... 24**

**Figure S25: ^13^C-NMR spectrum of compound 4a ................................................................25**

**Figure S26: Mass spectrum of compound 4a.................................................................... 26**

**Figure S27: IR spectrum of compound 4b. .......................................................................... 27**

**Figure S28: ^1^H-NMR spectrum of compound 4b................................................................. 28**

**Figure S29: ^13^C-NMR spectrum of compound 4b ................................................................ 29**

**Figure S30: Mass spectrum of compound 4b...................................................................... 30**

**Figure S31: IR spectrum of compound 5a. .......................................................................... 31**

**Figure S32: ^1^H-NMR spectrum of compound 5a................................................................. 32**

**Figure S33: ^13^C-NMR spectrum of compound 5a ................................................................ 33**

**Figure S34: IR spectrum of compound 5b. .......................................................................... 34**

**Figure S35: ^1^H-NMR spectrum of compound 5b................................................................. 35**

**Figure S36: ^13^C-NMR spectrum of compound 5b ............................................................... 36**

**Figure S37: IR spectrum of compound 5c. .......................................................................... 37**

**Figure S38: ^1^H-NMR spectrum of compound 5c................................................................. 38**

**Figure S39: ^13^C-NMR spectrum of compound 5c ................................................................ 39**

**
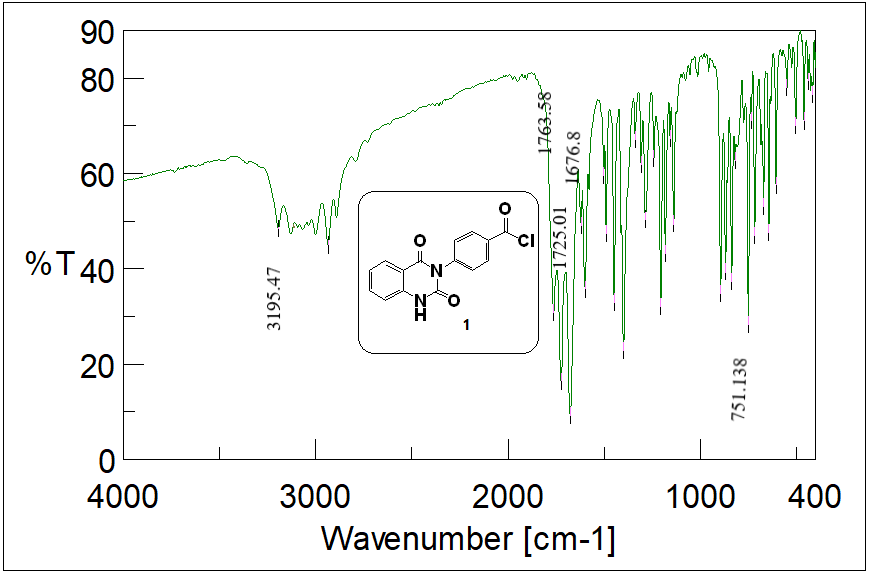
**

**Figure S1: - IR analysis of compound (1)**

**
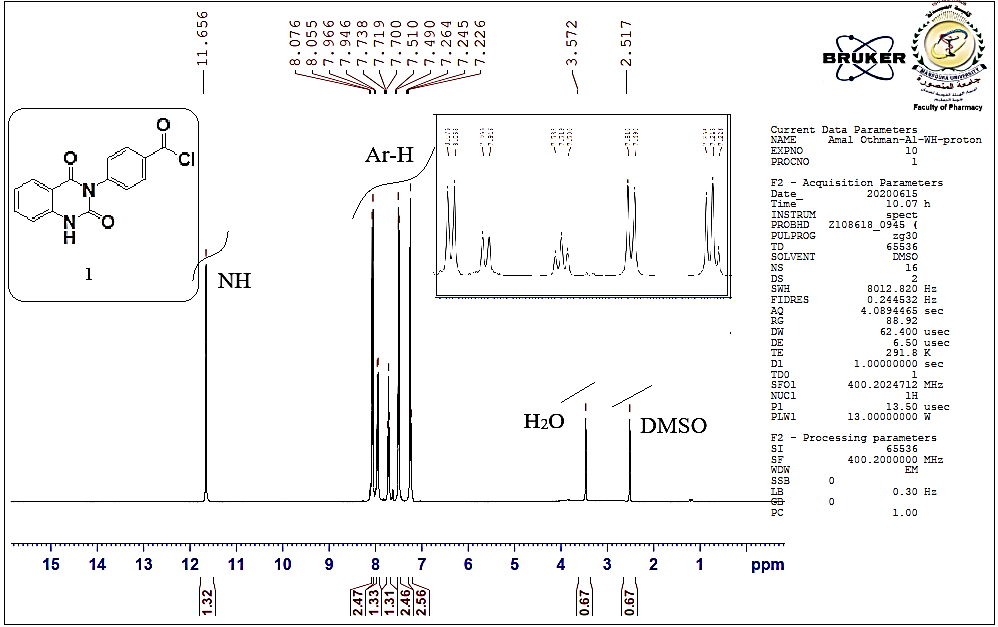
**

**Figure S2:- ^1^H-NMR analysis for compound (1)**

**
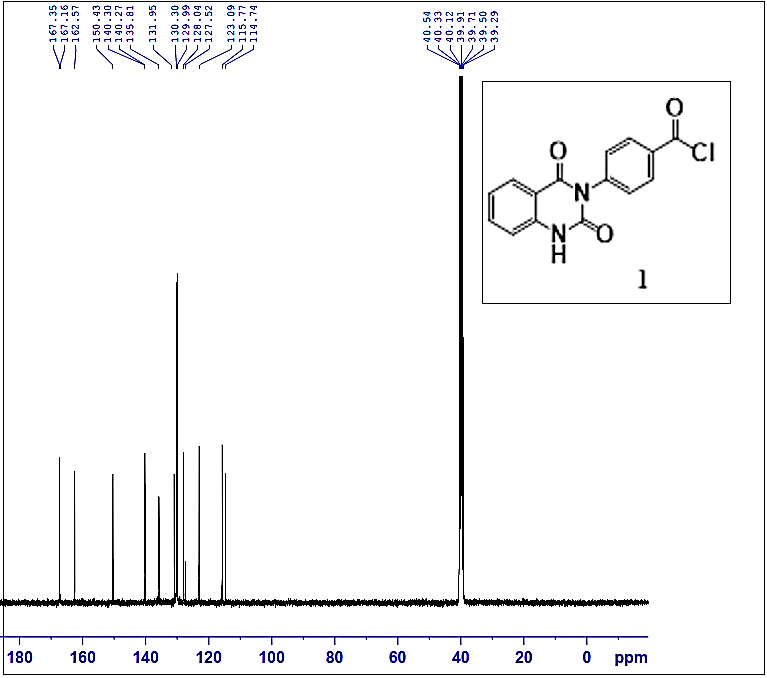
**

**Figure S3: - ^13^C-NMR analysis for compound (1)**


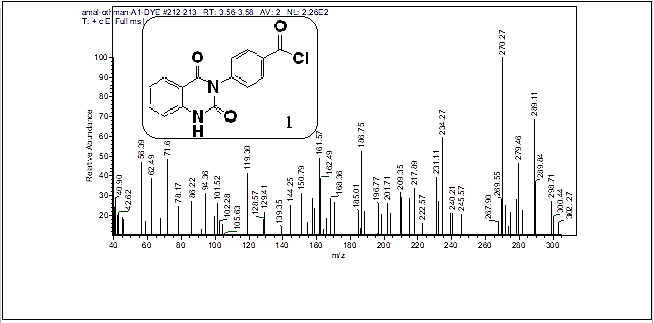


**Figure S4: - Mass analysis for compound (1)**


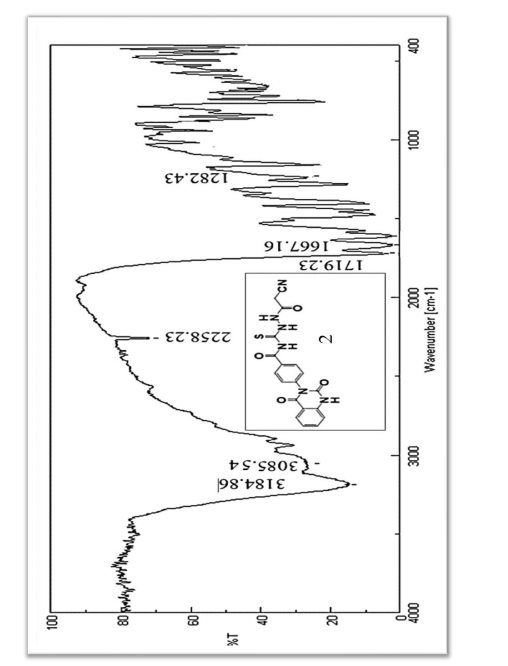


**Figure S5: IR spectrum of compound 2**


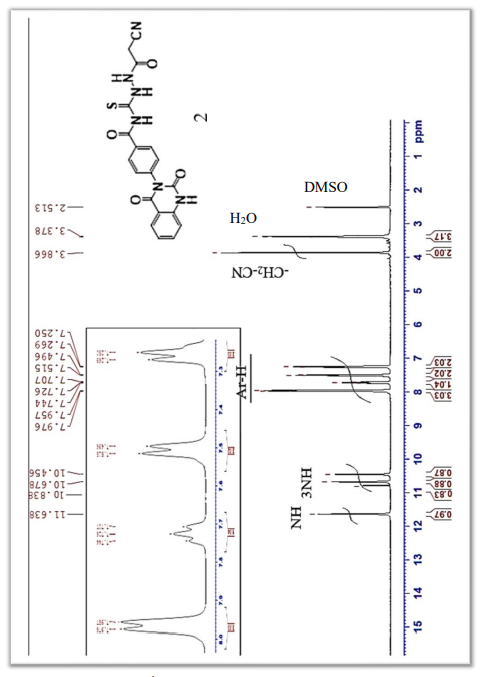


**Figure S6: ^1^H-NMR spectrum of compound 2**

**Figure S7: - ^13^C-NMR analysis for compound (2)
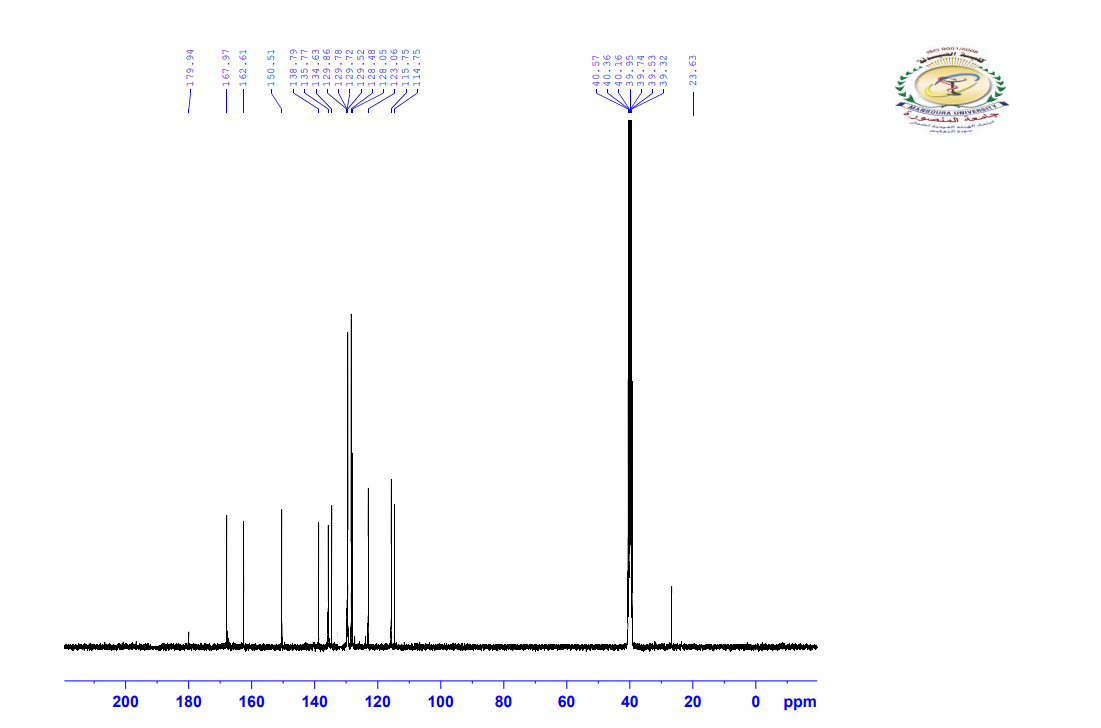
**


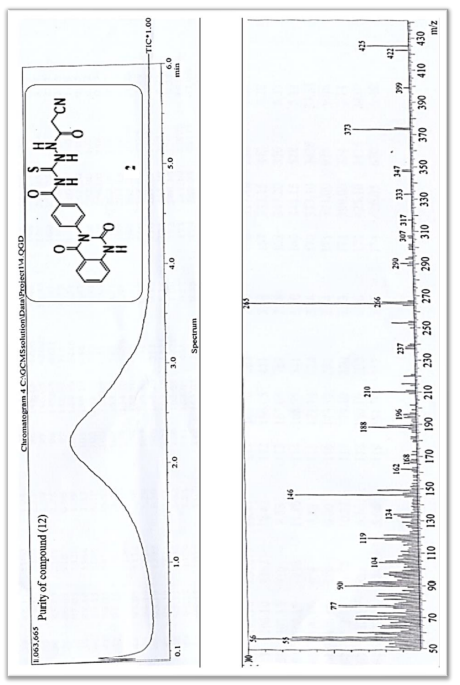


**Figure 8: Mass spectrum of compound 2**


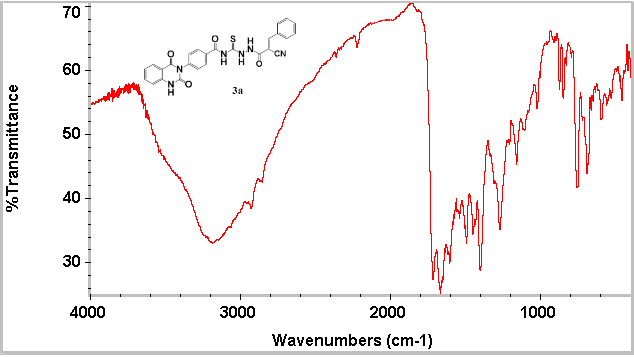


**Figure S9: IR spectrum of compound 3a**


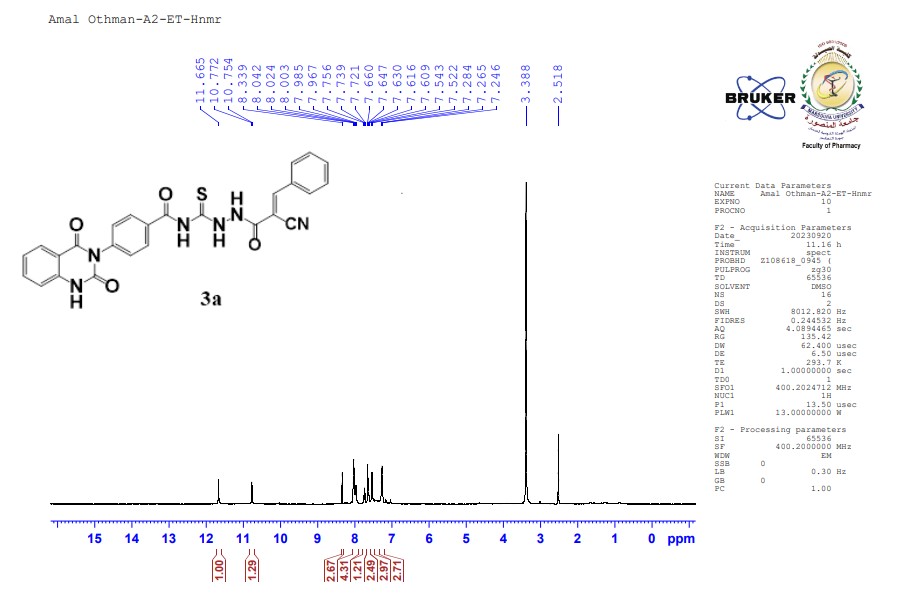


**Figure S10: ^1^H-NMR spectrum of compound 3a**

**Figure S11: ^13^C-NMR spectrum of compound 3a**


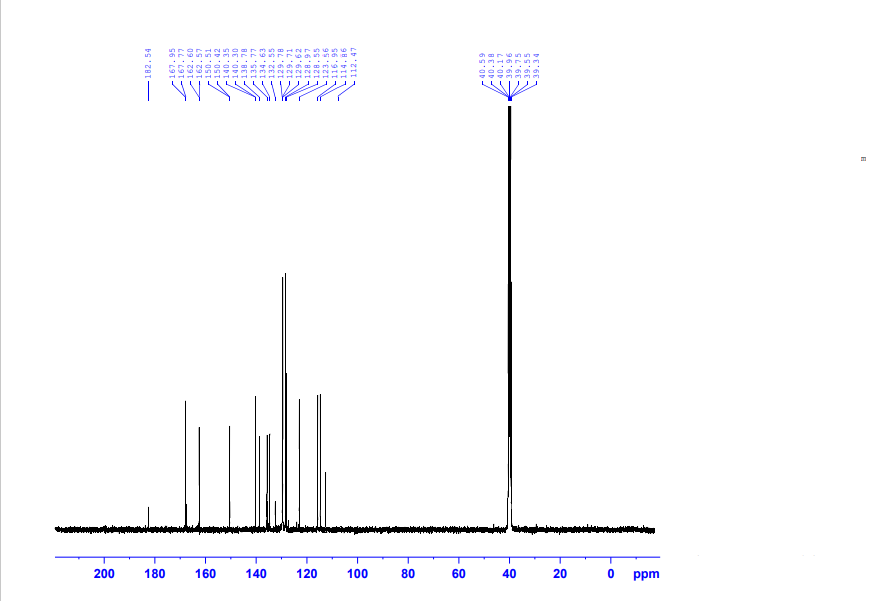


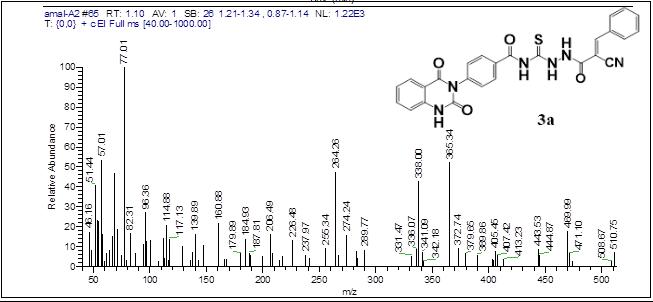


**Figure S12: Mass spectrum of compound 3a**


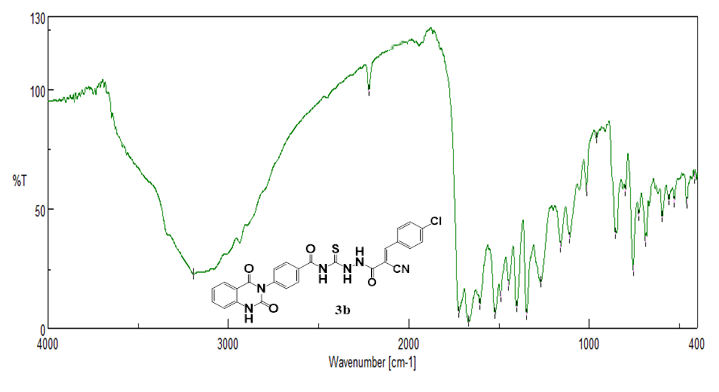


**Figure S13: IR spectrum of compound 3b**


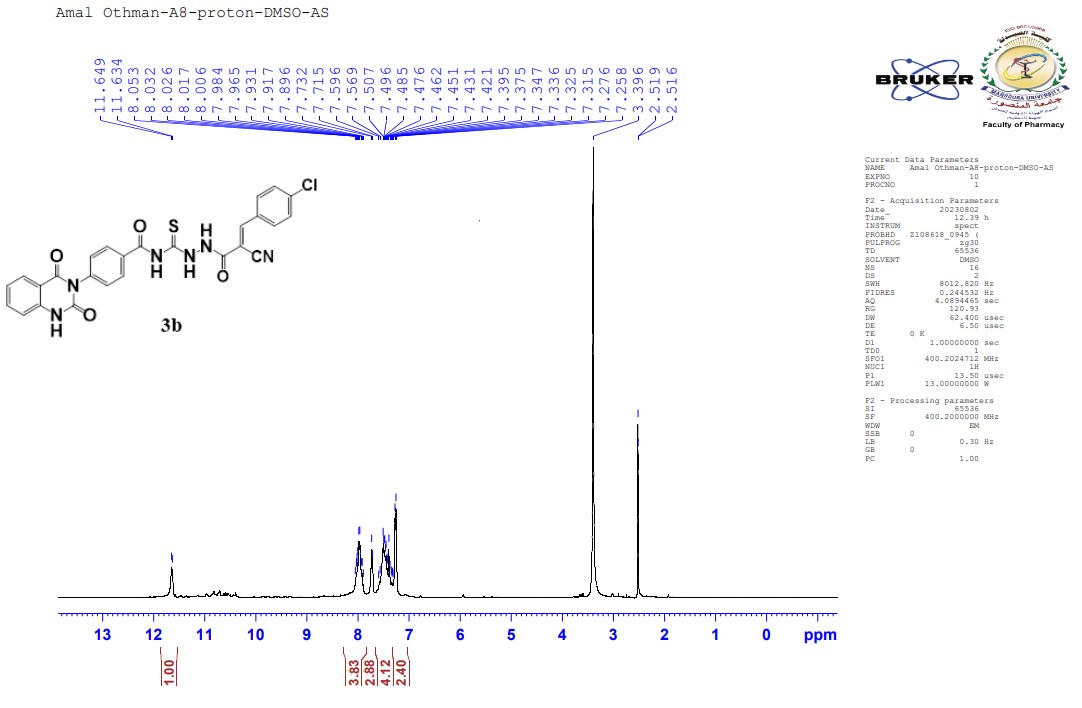


**Figure S14: ^1^H-NMR spectrum of compound 3b**


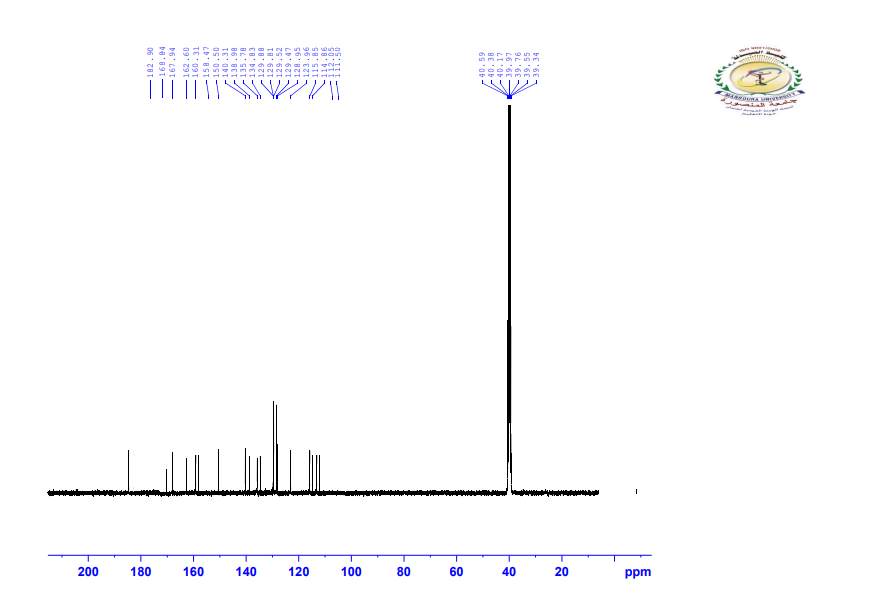


**Figure S15: ^1^H-NMR spectrum of compound 3b**


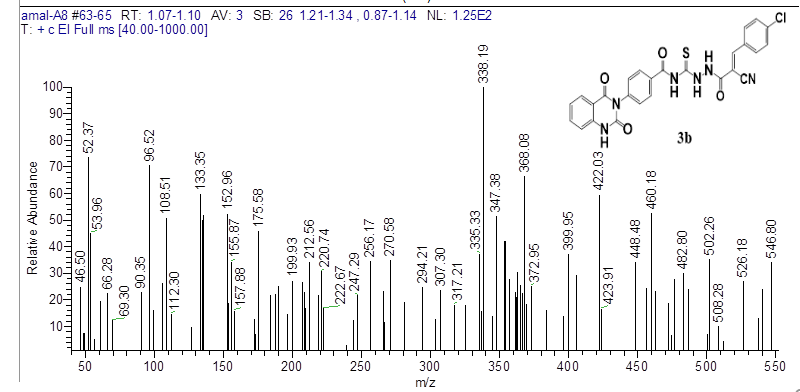


**Figure S16: Mass spectrum of compound 3b**


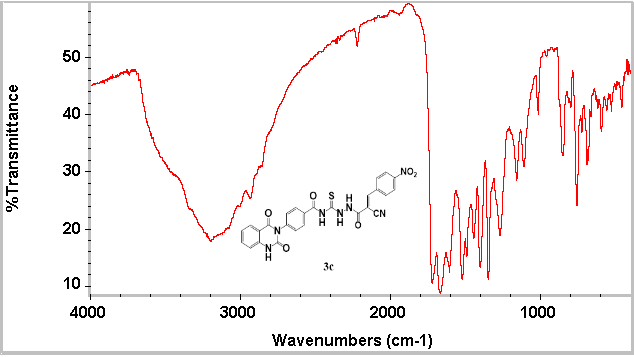


**Figure S17: IR spectrum of compound 3c**


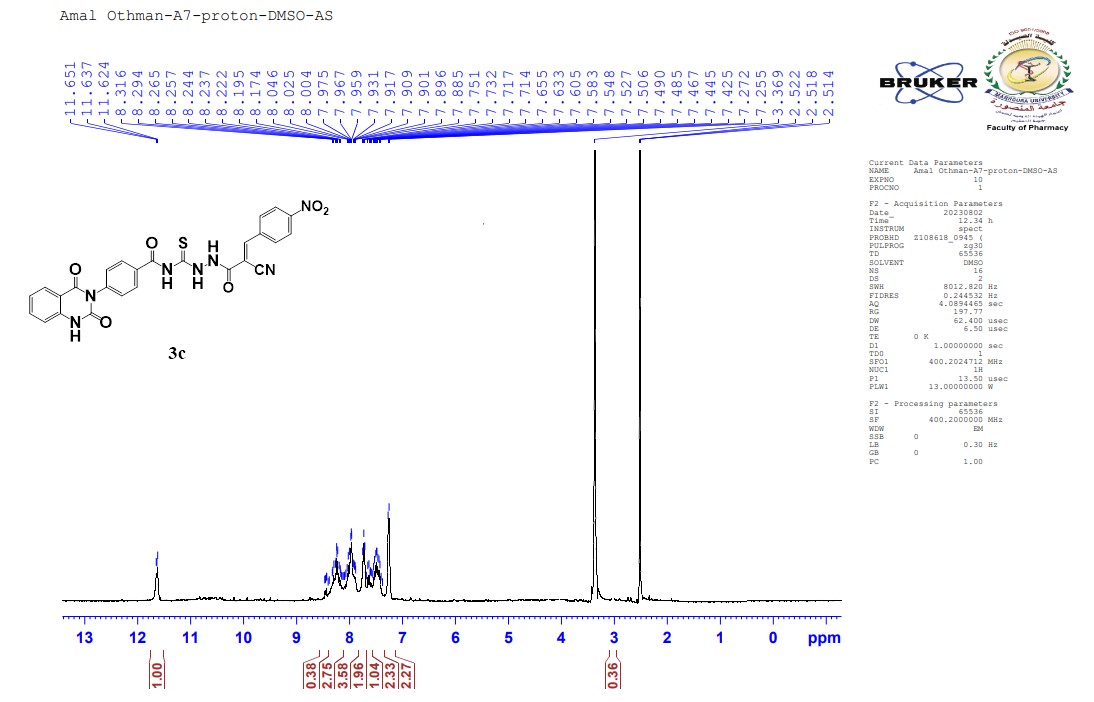


**Figure S18: ^1^H-NMR spectrum of compound 3c**


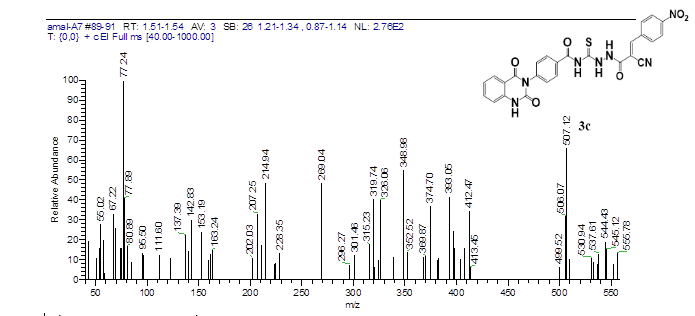


**Figure S19: Mass spectrum of compound 3c**


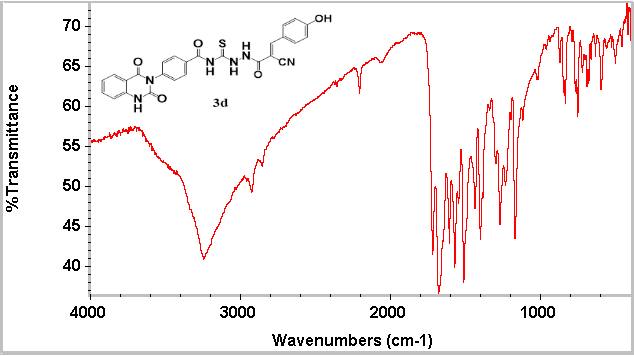


**Figure S20: IR spectrum of compound 3d**


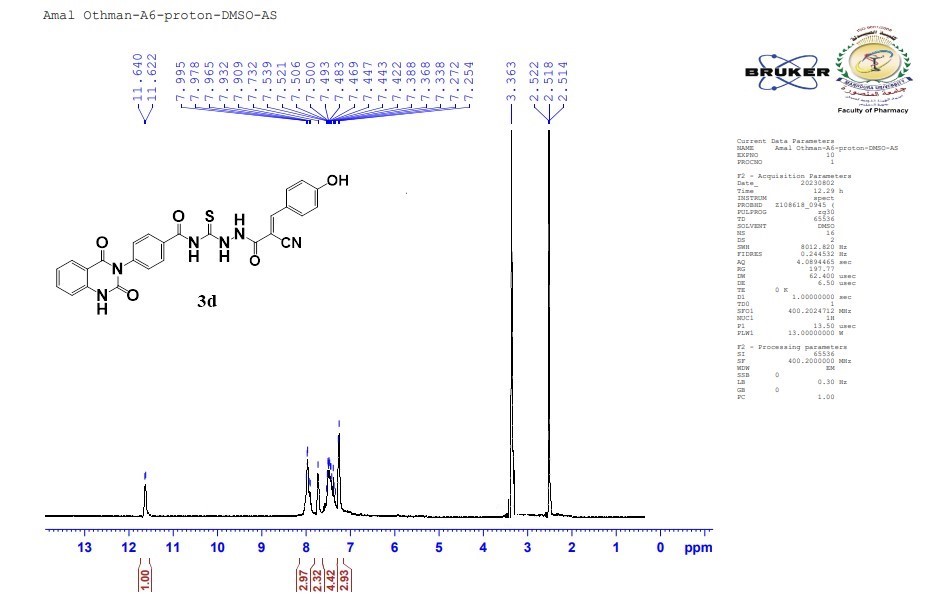


**Figure S21: ^1^H-NMR spectrum of compound 3d**


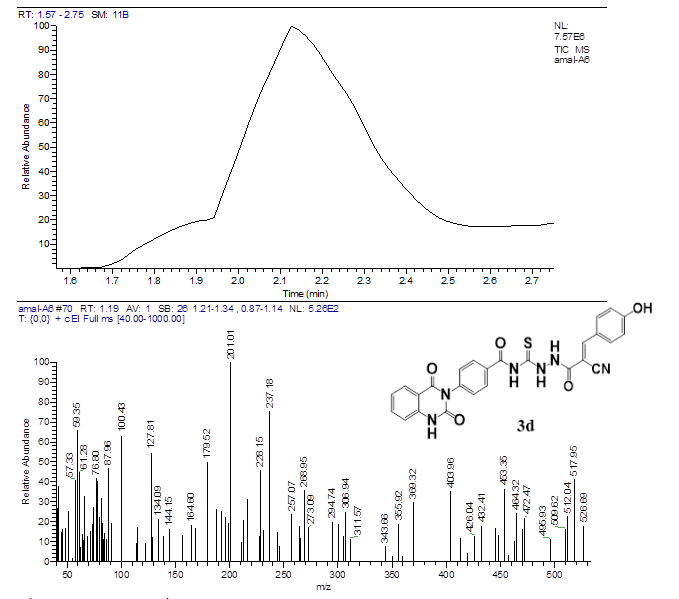


**Figure S22: Mass spectrum of compound 3d**


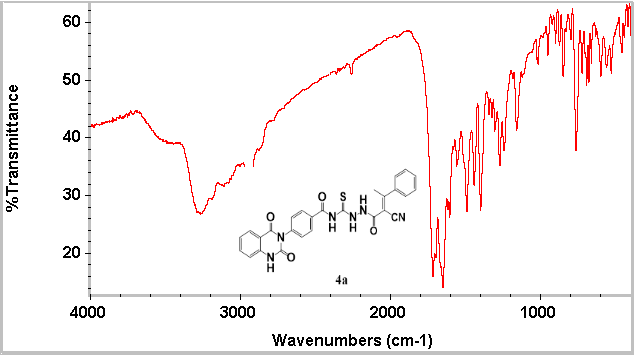


**Figure S23: IR spectrum of compound 4a**


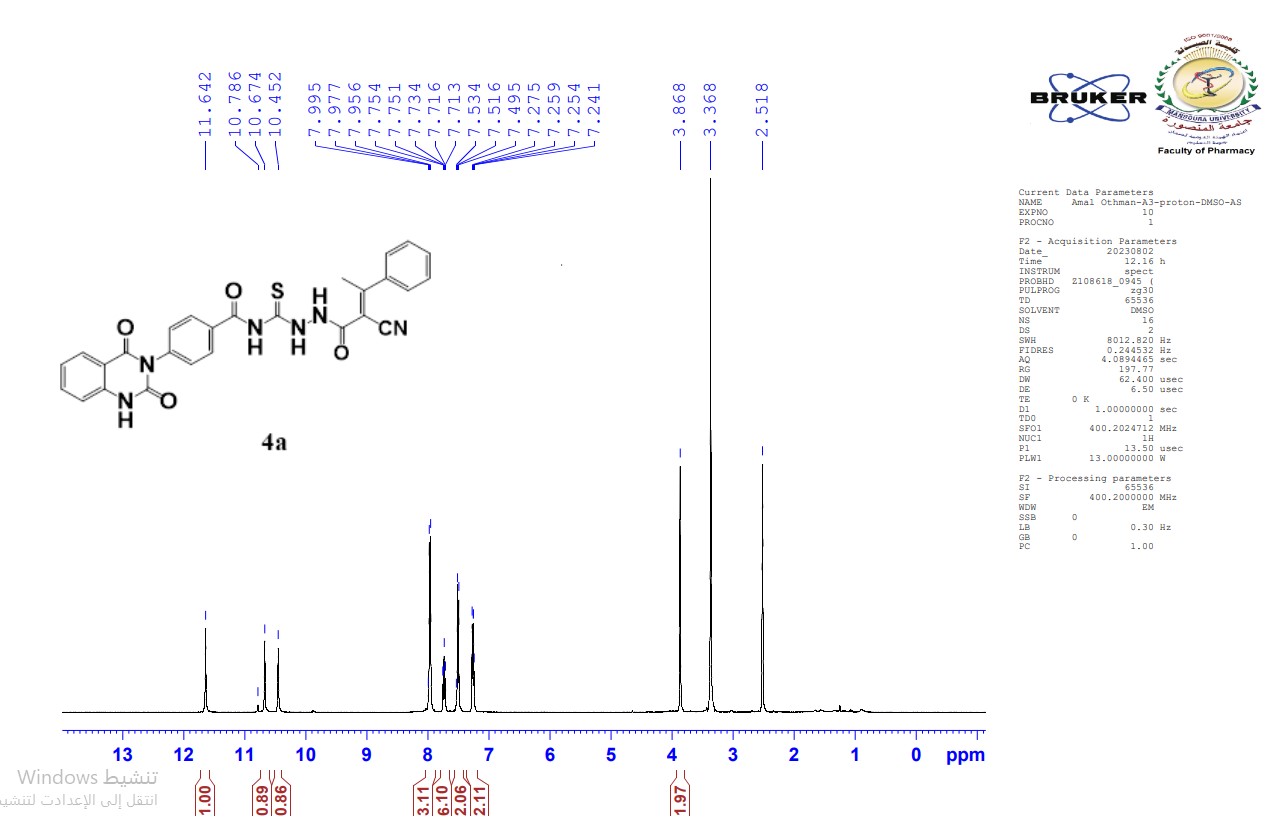


**Figure S24: ^1^H-NMR spectrum of compound 4a**


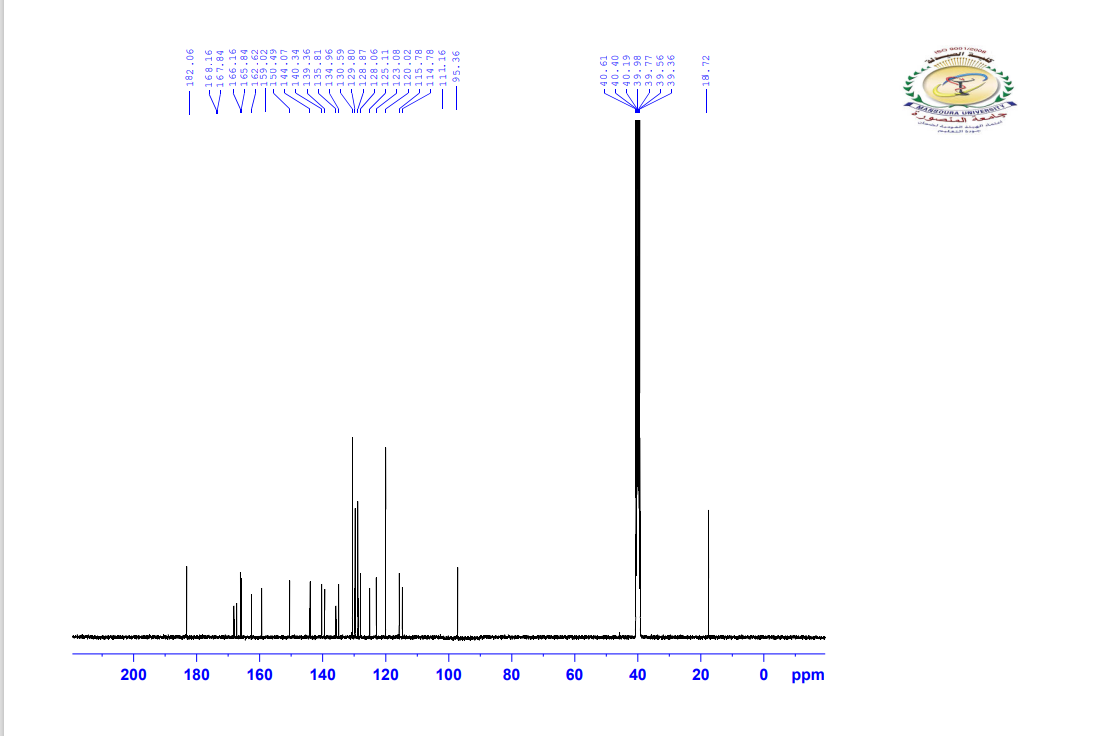


**Figure S25: ^13^C-NMR spectrum of compound 4a**


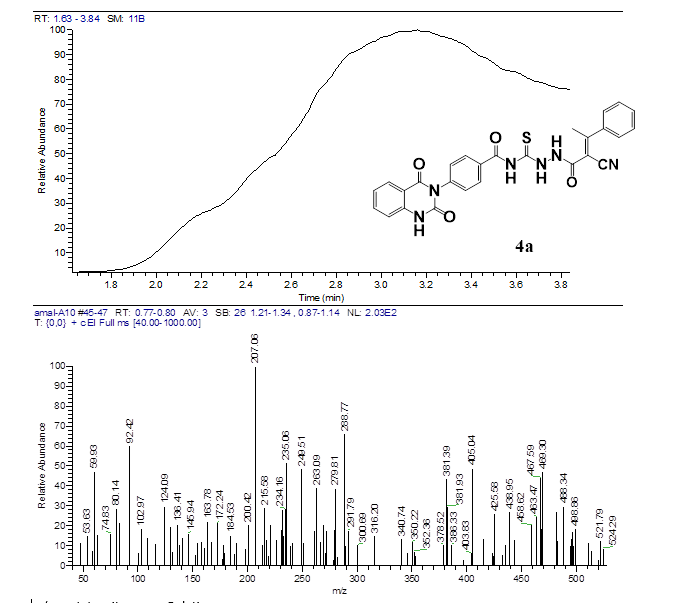


**Figure S26: Mass spectrum of compound 4a**


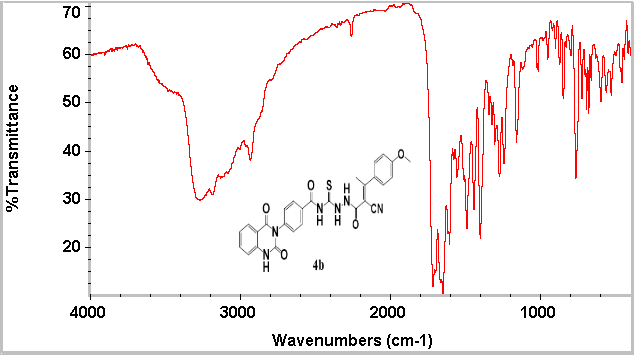


**Figure S27: IR spectrum of compound 4b**


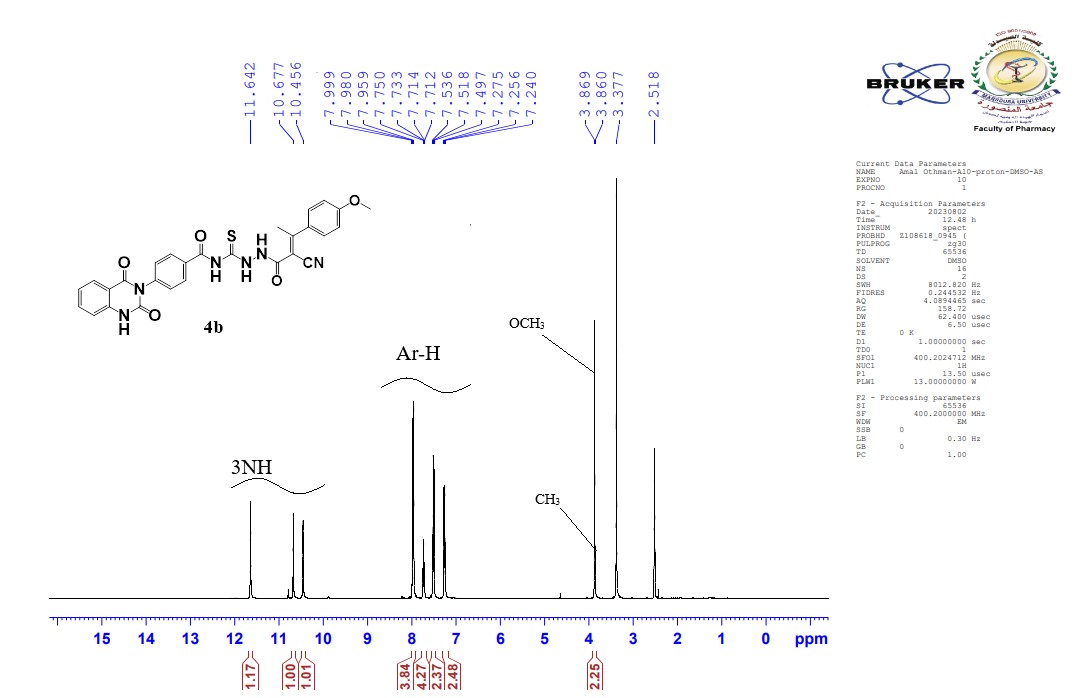


**Figure S28: ^1^H-NMR spectrum of compound 4b**

**Figure S29: ^13^C-NMR spectrum of compound 4b**


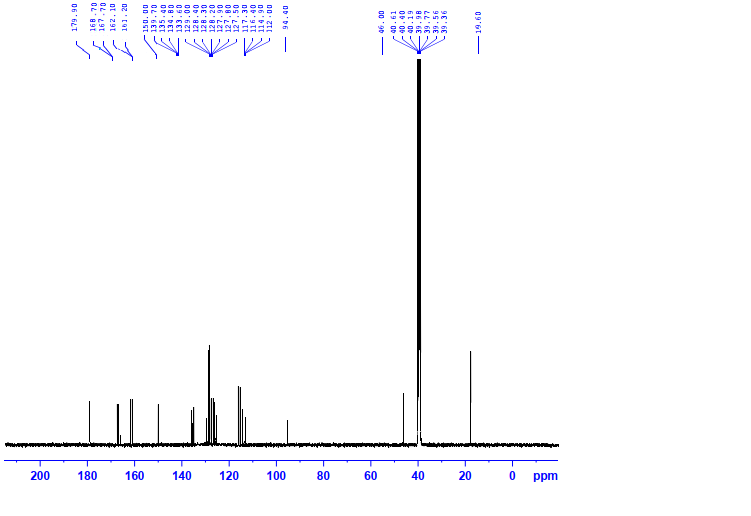


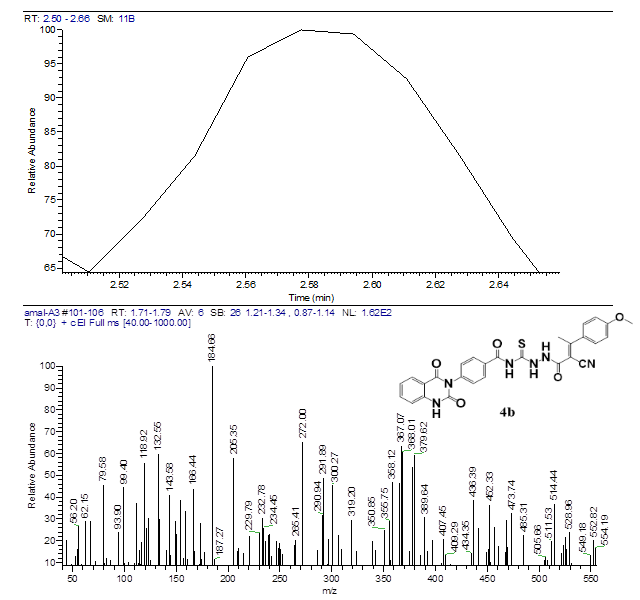
 **Figure S30: Mass spectrum of compound 4b**


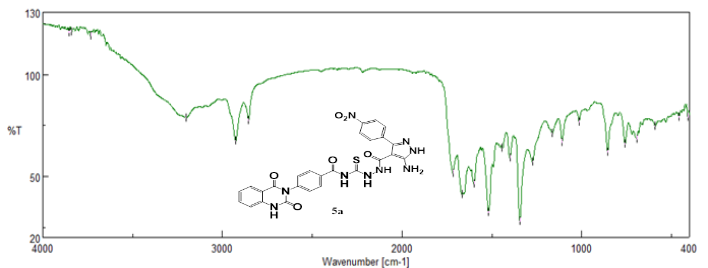


**Figure S31: IR spectrum of compound 5a**

**
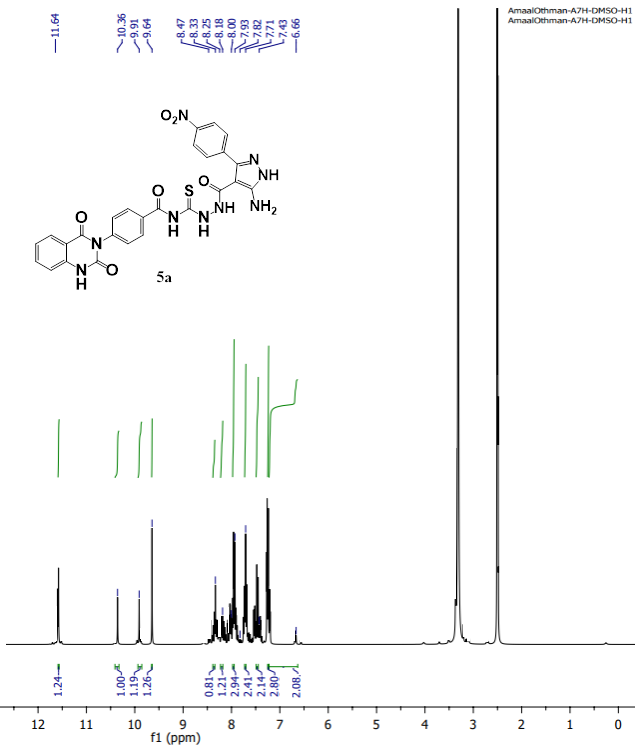
**

**Figure S32: ^1^H-NMR spectrum of compound 5a**

**Figure S33: ^13^C-NMR spectrum of compound 5a**


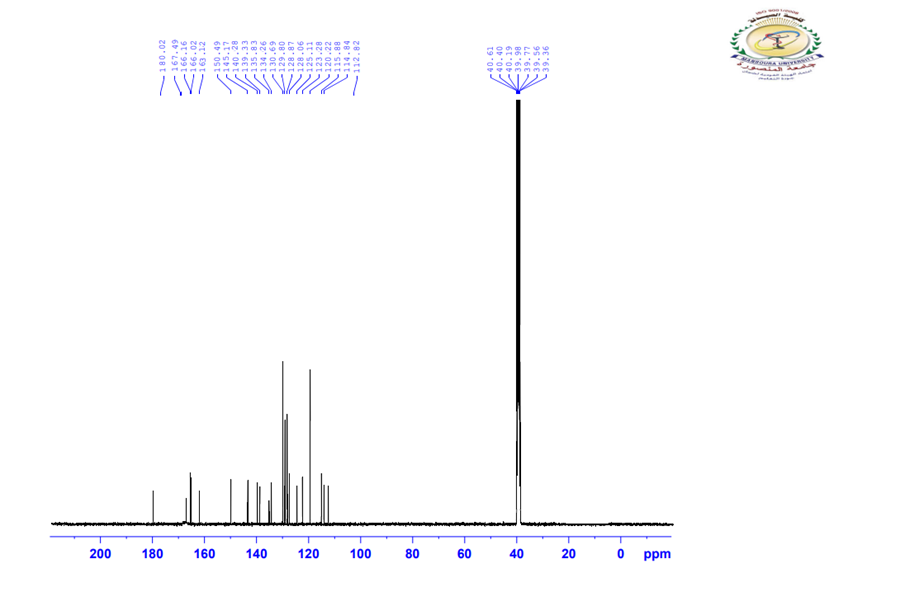


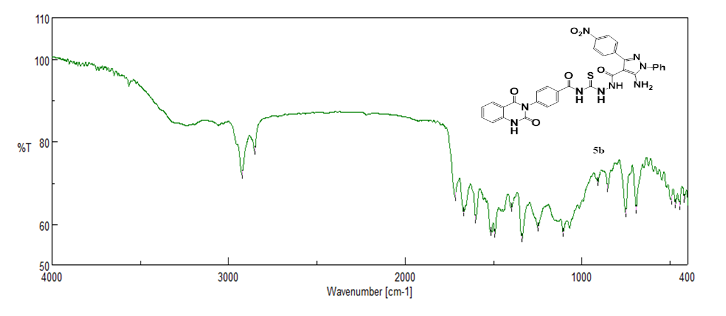


**Figure S34: IR spectrum of compound 5b**

**
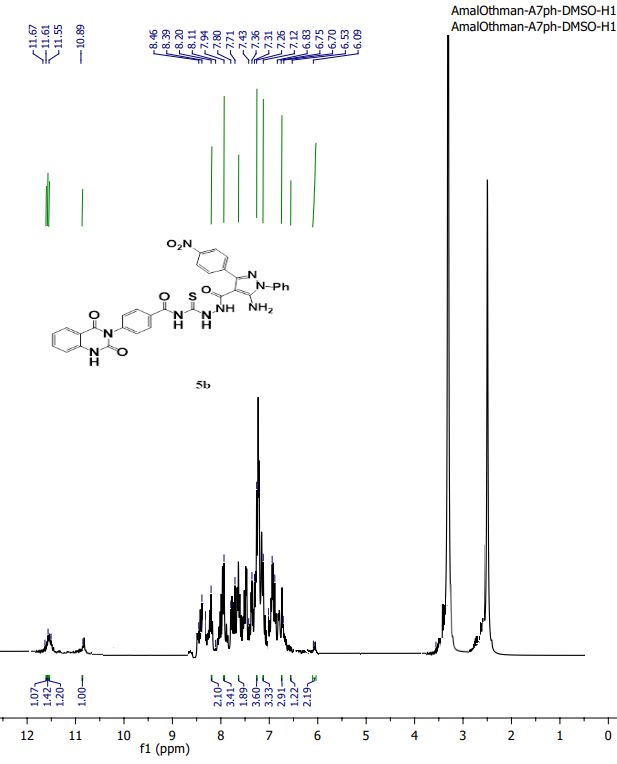
**

**Figure S35: ^1^H-NMR spectrum of compound 5b**

**Figure S36: ^13^C-NMR spectrum of compound 5b
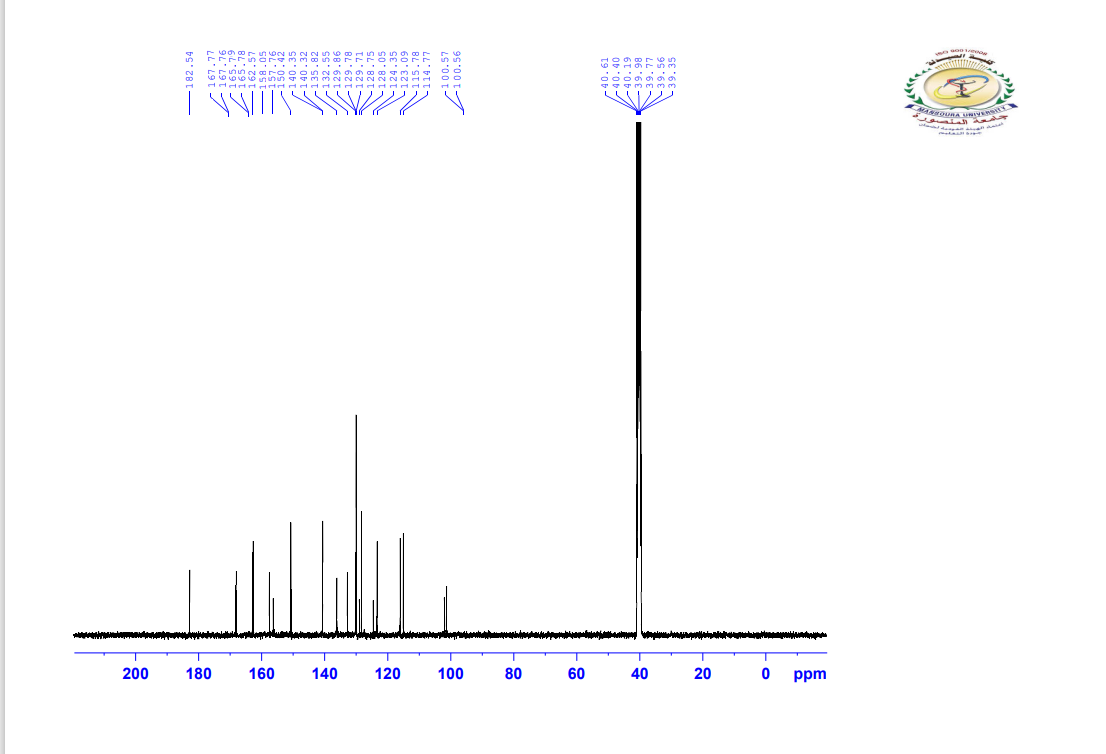
**


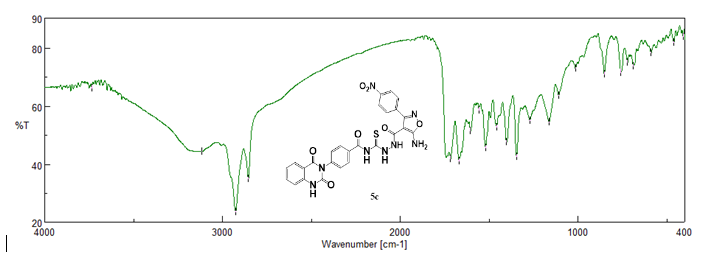


**Figure S37: IR spectrum of compound 5c**

**
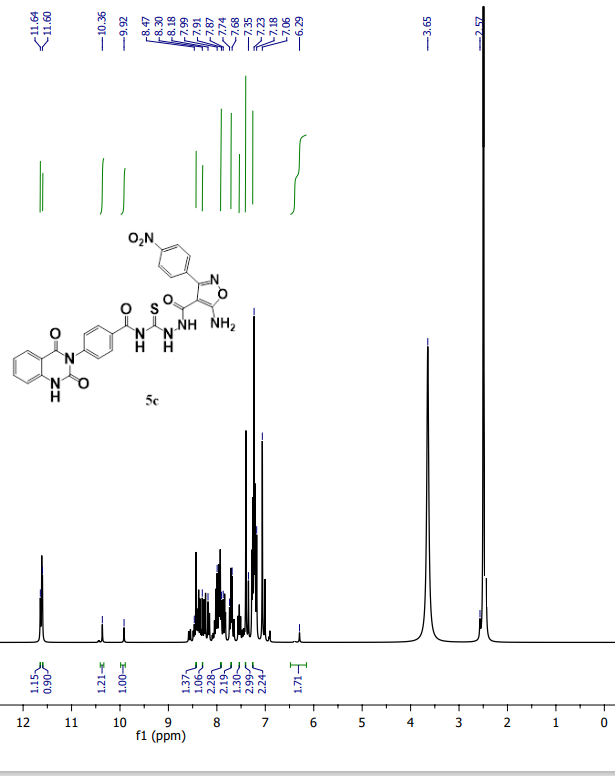
**

**Figure S38: ^1^H-NMR spectrum of compound 5c**

**
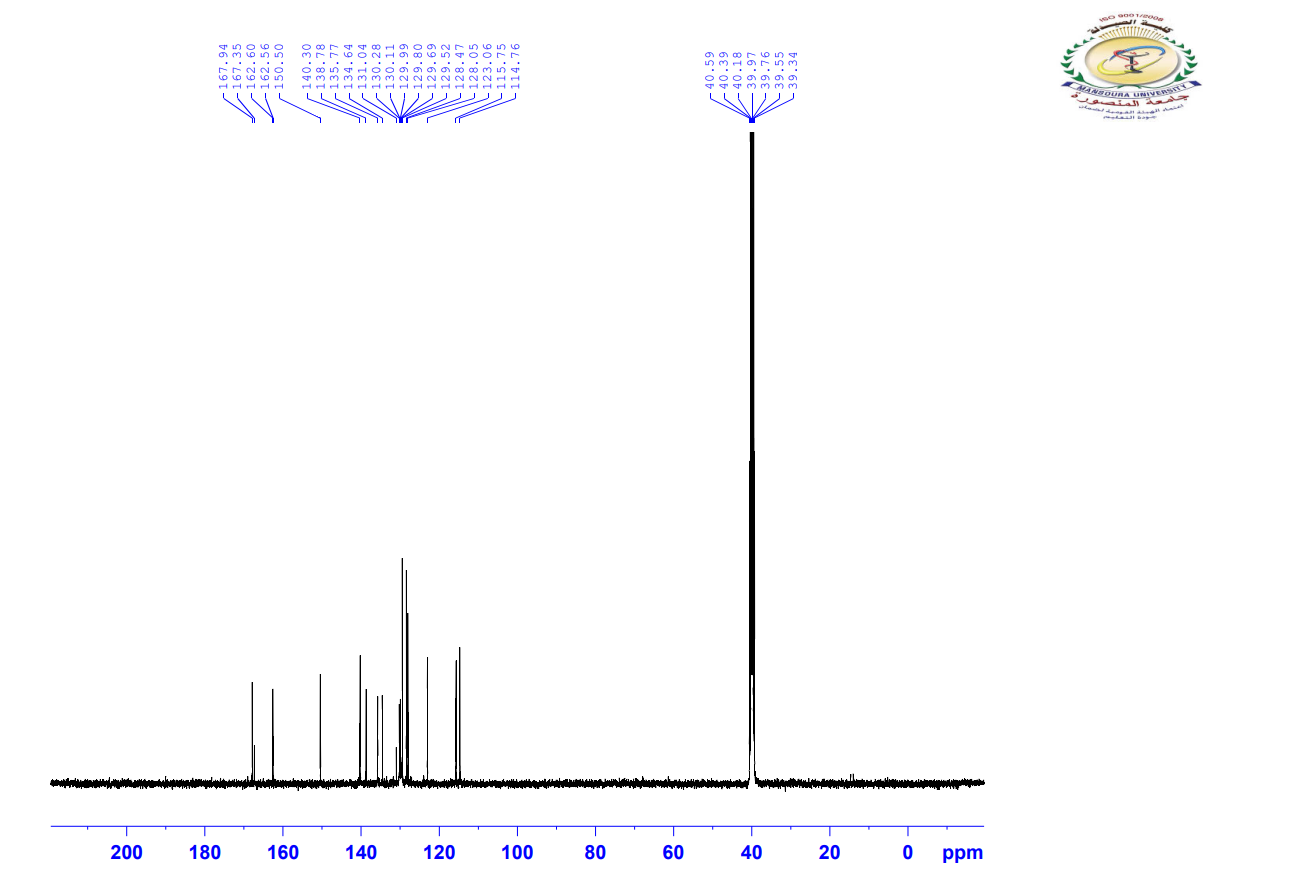
**

**Figure S39: ^13^C-NMR spectrum of compound 5c**
